# An Implantable Scaffold Sequentially Releasing STING Agonist and B7‐H3 Antibody for Bone Metastasis Immunotherapy

**DOI:** 10.1002/advs.202520642

**Published:** 2026-02-25

**Authors:** Qijun Lin, Hong Xiao, Shuai Fan, Kaimin Cai, Yanteng Xu, Xinwen Wang, Guanhong Chen, Chuandong Lang, Xinsheng Peng, Mingqiang Li, Yuhu Dai

**Affiliations:** ^1^ Department of Orthopedic Surgery The First Affiliated Hospital, Sun Yat‐sen University Guangzhou China; ^2^ Guangdong Provincial Key Laboratory of Orthopedics and Traumatology Guangzhou China; ^3^ Department of Medical Ultrasound, Laboratory of Novel Optoacoustic (Ultrasonic) Imaging The Third Affiliated Hospital Sun Yat‐sen University Guangzhou China; ^4^ Laboratory of Biomaterials and Translational Medicine, Center For Nanomedicine The Third Affiliated Hospital Sun Yat‐sen University Guangzhou China; ^5^ Department of Orthopedics The First Affiliated Hospital of USTC, Division of Life Sciences and Medicine University of Science and Technology of China Hefei Anhui China; ^6^ Cell‐Gene Therapy Center, Institute For Frontier Interdisciplinary Research in Health Sciences and Technology Sun Yat‐sen University Guangzhou China; ^7^ Key Laboratory for Polymeric Composite and Functional Materials of Ministry of Education Sun Yat‐sen University Guangzhou China

**Keywords:** bone metastasis, B7‐H3, controlled release, immunotherapy, MSA‐2, postoperative treatment

## Abstract

Immune checkpoint blockade (ICB) therapy has revolutionized cancer treatment, yet it remains largely ineffective against bone metastases. The immunosuppressive bone marrow microenvironment is a major barrier to ICB success in bone metastasis. Herein, we developed an implantable dual‐drug depot using a gelatin methacryloyl (GelMA) scaffold to prevent tumor recurrence and progression following surgical resection. The GelMA scaffold is co‐loaded with MSA‐2, a non‐nucleotide agonist of stimulator of interferon genes (STING), and calcium carbonate (CaCO_3_) microparticles (MPs) encapsulating B7‐H3 antibodies (αB7‐H3‐MPs). After implantation at the bone metastasis site, the scaffold provides sustained releases of MSA‐2 and αB7‐H3‐MPs. MSA‐2 is released first to activate the STING signaling pathway, triggering interferon secretion, reprogramming immunosuppressive cells, and promoting effector T cell infiltration and activation. Subsequently, dissolution of CaCO_3_ microparticles in the acidic tumor microenvironment facilitates the subsequent release of αB7‐H3, which blocks the B7‐H3 checkpoint upregulated by STING activation and prevents T‐cell exhaustion. This sequential release strategy was validated in multiple bone metastasis models, confirming its ability to produce a sustained and potent local antitumor immune response while reducing systemic toxicity associated with STING agonists and ICB drugs. Therefore, the scaffold^MSA‐2^_αB7‐H3‐MP_ represents a promising localized immunotherapy approach for the treatment of bone metastasis.

## Graphical abstract

We developed an implantable dual‐drug depot using GelMA for bone metastasis treatment, co‐delivering MSA‐2 and αB7‐H3‐loaded CaCO_3_ microparticles. Sustained release from GelMA scaffold enables MSA‐2 to activate STING signaling and enhance T‐cell infiltration and activation, while sequentially released αB7‐H3 blocks MSA‐2‐induced B7‐H3 upregulation, which prevents T‐cell exhaustion. This sequential release strategy offers a promising approach for bone metastasis treatment.

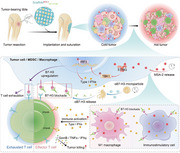

## 1 Introduction

Following the lungs and liver, bone is the third most common site for tumor metastasis [1]. The median survival after a bone metastasis is only about 1–3 years [2], underscoring the lethality of this condition. Standard treatment for bone metastases typically combines bone‐targeted therapies, including bisphosphonates, denosumab, and radium‐223, with systemic therapies like hormonal therapy or chemotherapy. However, these regimens generally yield suboptimal clinical outcomes [3]. Thus, novel therapeutic strategies for bone metastasis are urgently needed.

Immunotherapy, particularly immune checkpoint blockade (ICB), has revolutionized treatment outcomes in various malignancies. Given that bone marrow is a crucial immune organ rich in lymphocytes, there is great interest in leveraging immunotherapy against bone metastases [4]. However, larger clinical studies indicate that bone metastases generally respond poorly to ICB therapy [5, 6]. This paradox is attributed to the bone marrow's dual immune nature: it harbors not only effector lymphocytes but also abundant immunosuppressive cells like myeloid‐derived suppressor cells (MDSCs) [7]. When tumor cells colonize the bone marrow, these immunosuppressive cells form an “immune barrier” around the tumor, preventing infiltration by cytotoxic immune cells such as T cells and natural killer (NK) cells [8, 9]. As a result, bone metastases are classified as “cold” tumors characterized by a paucity of active T cells, and converting this microenvironment to a “hot” one is essential for ICB efficacy.

Activation of the stimulator of interferon genes (STING) pathway has recently gained attention as a strategy to turn “cold” tumors “hot”. STING agonists can trigger signaling transduction and produce downstream type I interferons (especially IFNβ) [10]. They have been shown to activate antigen‐presenting dendritic cells (DCs), reprogram immunosuppressive tumor‐associated macrophages, limit the accumulation of inhibitory MDSCs, and promote the recruitment and activation of CD8^+^ T cells and NK cells [11], thereby creating conditions favorable for ICB therapy. The conventional STING agonists cyclic dinucleotide (CDNs) suffer from poor bioavailability because their high hydrophilicity limits cell membrane penetration, and they are readily degraded by nucleases and phosphodiesterase in the tumor microenvironment (TME) [12]. By contrast, MSA‐2 is an innovative non‐nucleotide small‐molecule STING agonist [13], that has demonstrated robust efficacy in multiple studies [14, 15]. Under acidic TME conditions, the carboxyl group of MSA‐2 becomes protonated and more hydrophobic, facilitating its transmembrane transport and cellular uptake. Nonetheless, MSA‐2's short half‐life and transient duration of action limit its therapeutic window [13]. Furthermore, STING agonists have been reported to upregulate immune checkpoint molecules in the TME [14, 16], potentially resulting in adaptive immune resistance.

Tumors that predominantly metastasize to bone, such as prostate cancer (PCa), respond poorly to conventional ICB agents targeting PD‐1 or CTLA‐4 [17, 18]. B7‐H3 (CD276), a member of the B7 superfamily of co‐regulatory molecules, has emerged as a promising alternative immune checkpoint target in these contexts. Unlike PD‐L1, which is often only weakly expressed in tumors, B7‐H3 is highly expressed in metastatic and advanced cancers [19, 20], and minimally expressed in normal tissues [20], making it an attractive target for immunotherapy. Several clinical trials of B7‐H3‐targeted therapies are currently underway, with preliminary results indicating encouraging safety and efficacy profiles [20], paving the way for therapeutic antibodies against B7‐H3.

A systemic administration of STING agonists or ICBs risks a broad spectrum of immune‐related adverse effects [21, 22]. Compounding these issues, the unique vascularization of bone often results in subtherapeutic drug concentrations in the bone marrow following systemic delivery [23]. Local drug delivery offers a compelling solution to these challenges. Notably, when bone metastases cause complications like spinal cord compression or pathological fracture, surgical intervention is often required. Such surgeries provide an opportunity to implant a localized drug depot at the resection site, ensuring sustained antitumor immune activity while minimizing off‐target effects and systemic toxicity. An ideal local delivery system should strongly adhere to the tumor site, be biocompatible, and provide controlled, sustained release of therapeutic agents. Various biomaterials, including collagen, chitosan, and gelatin, have been successfully used to construct local drug‐releasing scaffolds [24, 25, 26, 27]. Among these, gelatin methacryloyl (GelMA) hydrogels stand out due to their tunable morphology and mechanical properties, and excellent biocompatibility. Furthermore, freeze‐dried GelMA forms a porous, adhesive structure well‐suited for implantation and prolonged drug release [28], effectively extending the duration of therapy in the tumor site.

**SCHEME 1:**
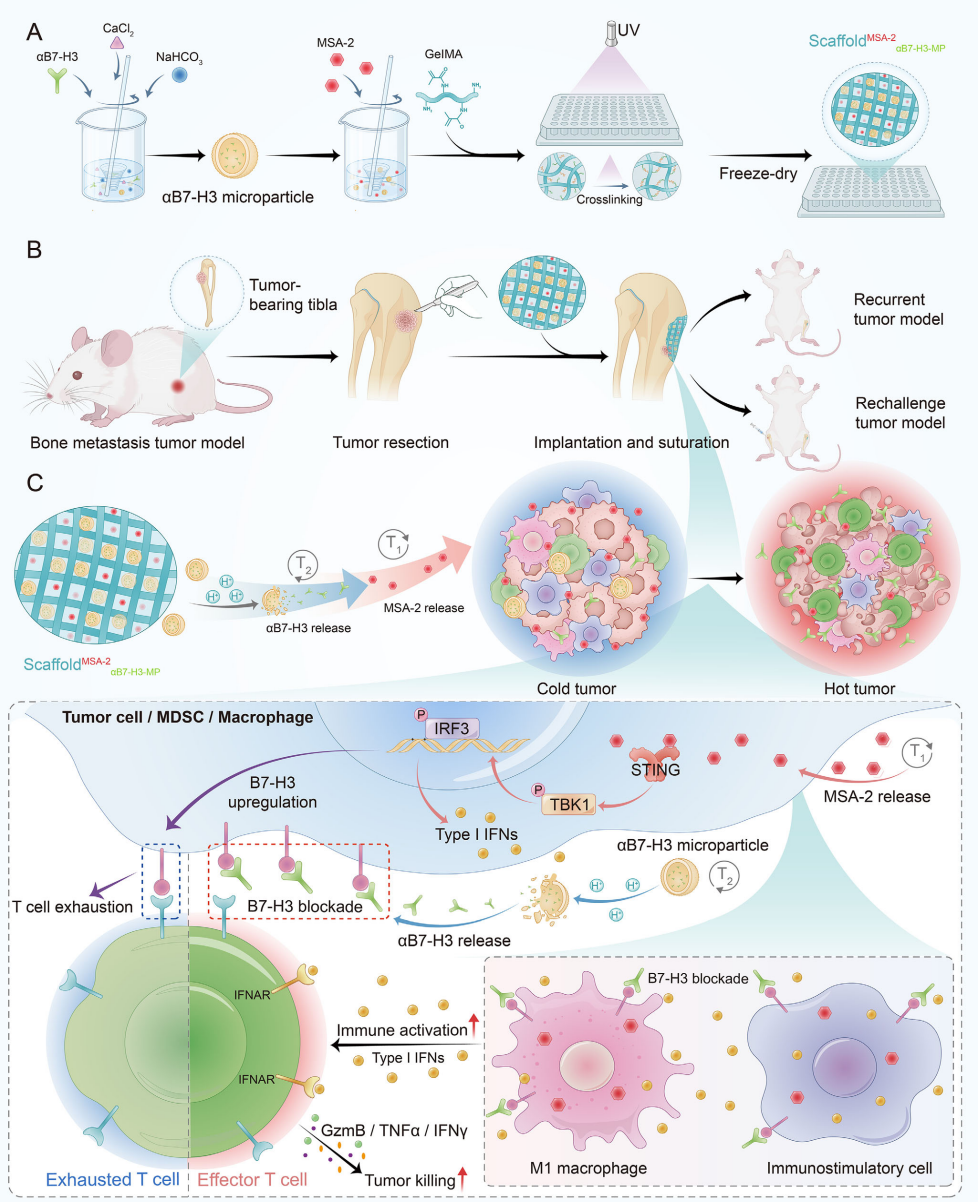
Schematic illustration of GelMA scaffold incorporating αB7‐H3‐loaded calcium carbonate microparticles (αΒ7‐H3 microparticle) and the STING agonist MSA‐2. A) Fabrication process of the GelMA scaffold loaded with αB7‐H3 microparticles and MSA‐2. B) Postoperative application: following tumor resection with positive margins, the dual‐drug scaffold is implanted at the resection site to enable sustained and localized drug release. C) Therapeutic mechanism: synergistic activation of STING signaling and blockade of B7‐H3 promotes robust antitumor immunity, preventing postoperative tumor recurrence.

Herein, we report an implantable dual‐drug GelMA scaffold co‐loaded with the STING agonist MSA‐2 and an anti‐B7‐H3 antibody (αB7‐H3) encapsulated in CaCO_3_ microparticles (αB7‐H3‐MPs). This scaffold is designed to synergistically elicit a strong and long‐lasting local antitumor immune response to prevent postoperative tumor recurrence in bone (Scheme 1). We prepared αB7‐H3‐MPs by a controlled CaCO_3_ precipitation process in the presence of αB7‐H3, then embedded these microparticles into GelMA hydrogel along with MSA‐2. After UV crosslinking and lyophilization, the resulting porous GelMA scaffold can be implanted in situ at the tumor resection site. As the scaffold gradually degrades, it continuously releases MSA‐2 and αB7‐H3‐MPs into the local bone microenvironment. MSA‐2 is released first to activate STING signaling in MDSCs, tumor‐associated macrophages (TAMs), and tumor cells, thereby inducing type I interferon production and promoting the recruitment and activation of effector T cells to attack residual tumor cells. Subsequently, the CaCO_3_ microparticles gradually dissolve under acidic TME conditions to release αB7‐H3, which blocks the upregulated B7‐H3 immune checkpoint and prevents T cell exhaustion. By design, this sequential release strategy ensures that STING activation primes the tumor microenvironment for effective checkpoint blockade, while delayed αB7‐H3 release counteracts STING‐induced B7‐H3 expression in tumor and immune cells. Overall, our engineered scaffold demonstrated potent antitumor efficacy in multiple bone metastasis models and offers a promising localized immunotherapeutic strategy to treat bone metastases.

## 2 Results

### 2.1 Fabrication and Characterization of MSA‐2 and αB7‐H3 Microparticle‐Loaded GelMA Scaffold

**FIGURE 1:**
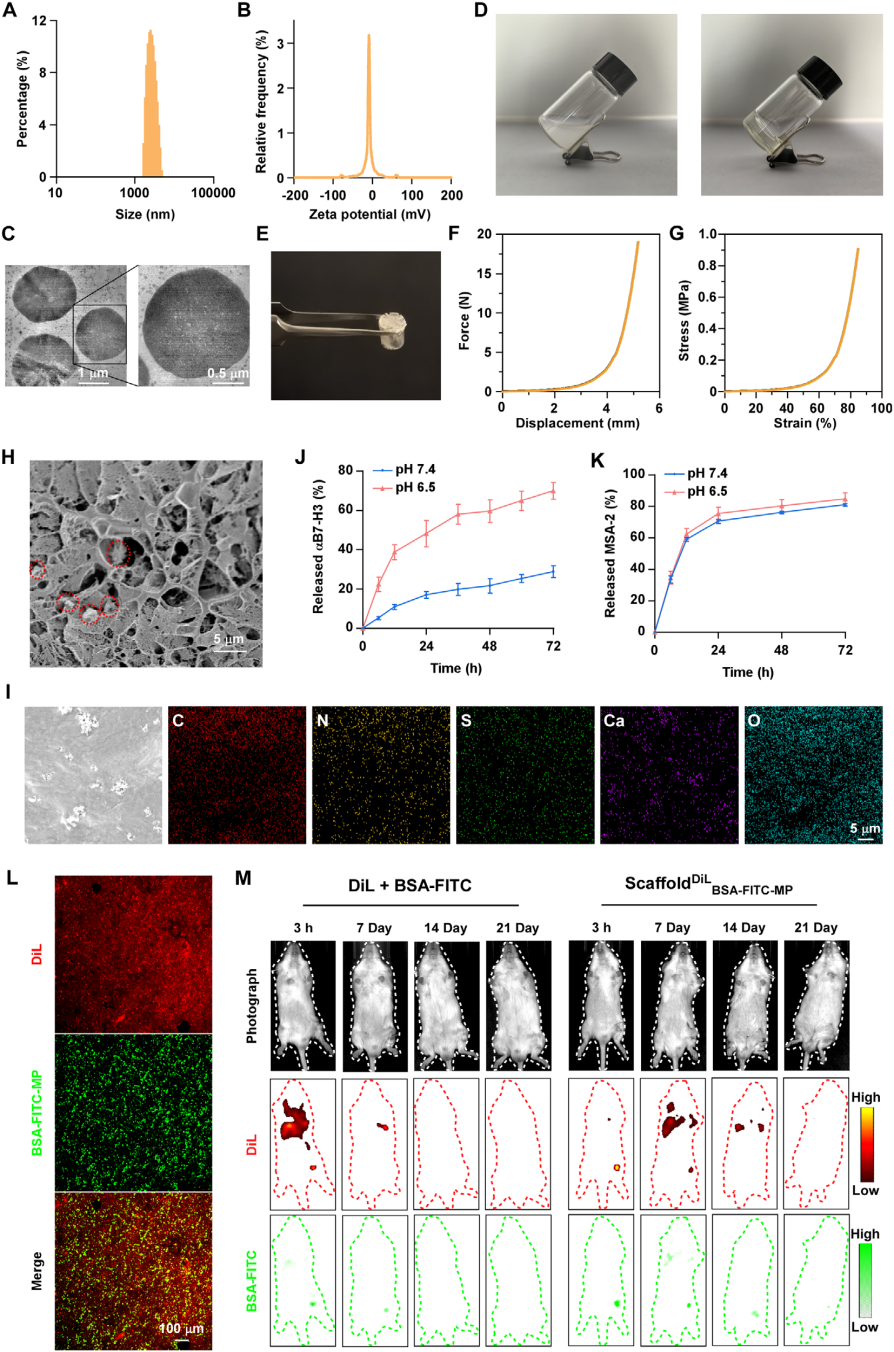
Characterization of the scaffold^MSA‐2^_αB7‐H3‐MP_ composite. A, B) Particle size distribution (A) and zeta potential (B) of αB7‐H3‐MPs. C) TEM images of αB7‐H3‐MPs. D) Photographs of the scaffold before (left) and after (right) UV photo‐crosslinking. E) Image of freeze‐dried scaffold. F, G) Maximum compressive strength (F) and compressive modulus (G) of the scaffold. H) SEM images of the scaffold, with red circles indicating embedded αB7‐H3‐MPs. I) Elemental mapping (C, N, S, Ca, and O) of the GelMA by EDS. J, K) In vitro cumulative release profiles of αB7‐H3 (J) and MSA‐2 (K) at pH 7.4 and 6.5. L) Confocal fluorescence microscopy images of BSA‐FITC‐MPs and DiL‐loaded scaffold. Scale bar, 100 µm. M). In vivo drug release behavior in a bone metastatic tumor model monitored by fluorescence imaging.

αB7‐H3 microparticles (αB7‐H3‐MPs) were synthesized via a controlled chemical precipitation method. Dynamic light scattering (DLS) measurements showed an average hydrodynamic diameter of 2.92 ± 0.76 µm and a zeta potential of −9.22 ± 1.26 mV (Figure 1A, B). The transmission electron microscope (TEM) confirmed the microparticles' spherical morphology with an average diameter of approximately 2 µm (Figure 1C), consistent with DLS results. Enzyme‐linked immunosorbent assay (ELISA) further indicated that the loading content of the B7‐H3 antibody was about 2.48% (w/w) in these microparticles.

Next, we embedded the αB7‐H3‐MPs and the STING agonist MSA‐2 into a biocompatible GelMA hydrogel matrix to create a composite material. This composite was photo‐crosslinked by UV exposure to form a stable hydrogel, ensuring prolonged retention at the tumor resection site (Figure 1D). This hydrogel composite was then lyophilized to yield a porous scaffold suitable for in situ implantation (Figure 1E). Mechanical characterization of the scaffold showed a maximum compressive strength of 19.00 N and a compressive modulus of 5.47 MPa (Figure 1F,G). Scanning electron microscopy (SEM) revealed a highly porous architecture with uniformly distributed αB7‐H3‐MPs embedded throughout the scaffold (Figure 1H). In addition, energy‐dispersive X‐ray spectroscopy (EDS) elemental mapping confirmed the successful integration of αB7‐H3‐MPs within the GelMA scaffold (Figure 1I).

We then evaluated the release profiles of the two therapeutic components under physiological vs tumor‐mimicking conditions. Under neutral pH 7.4, the scaffold released only ∼28% of the loaded αB7‐H3 over 72 h; whereas under acidic pH 6.5 (simulating the tumor microenvironment), it released ∼70% in the same period (Figure 1J). By contrast, MSA‐2 exhibited a pH‐independent release, with nearly overlapping release curves at pH 7.4 and 6.5 (Figure 1K). To visualize the distribution and co‐localization of the payloads in the hydrogel, we prepared model constructs using fluorescein‐labeled bovine serum albumin (BSA‐FITC) in place of αB7‐H3 (loaded into CaCO_3_) and a fluorescent dye (DiL) as a surrogate for MSA‐2. Confocal fluorescence microscopy images of the resulting BSA‐FITC‐MPs & DiL‐loaded GelMA hydrogel showed homogeneous green and red fluorescence throughout the scaffold, confirming that both payloads were uniformly distributed and co‐encapsulated (Figure 1L).

Finally, we assessed the in vivo release and retention of the drugs using a bone metastasis tumor model. In vivo fluorescence imaging revealed that free BSA‐FITC and DiL (delivered without a scaffold) were rapidly cleared from the implantation site within 7 days. In contrast, the GelMA scaffold loaded with BSA‐FITC‐MPs and DiL maintained a strong local fluorescent signal for at least 14 days (Figure 1M; Figure S1), indicating sustained drug release at the target site. Overall, this pH‐sensitive delivery system achieves a sequential and prolonged release of immunomodulatory agents in the acidic TME, offering a clear advantage for localized immunotherapy of bone metastatic cancer.

### 2.2 In Vitro Analysis of STING Pathway Activation and Immunosuppressive Cell Reprogramming

**FIGURE 2:**
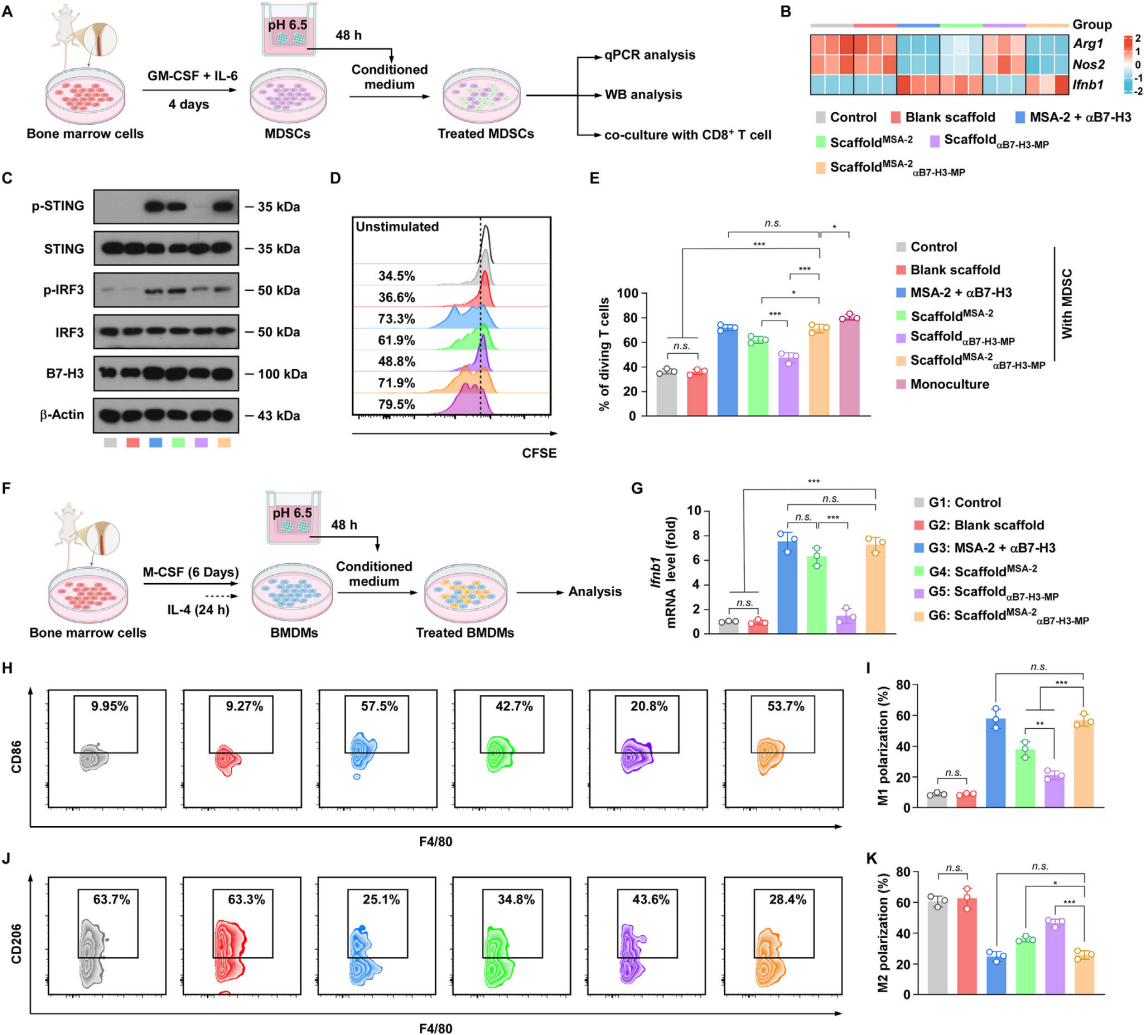
In vitro analysis of STING pathway activation and the immune modulation induced by the drug‐loaded GelMA scaffold. A) Schematic illustration of MDSC induction, culture, treatment, and analysis. B) The heatmap of *Arg1*, *Nos2*, and *Ifnb1* mRNA expression in MDSCs after 6 h of treatment (*n* = 3 independent experiments). C) Representative western blots showing STING pathway proteins (STING, p‐STING, IRF3, p‐IRF3) and B7‐H3 expression in MDSCs. D, E) Representative CFSE dilution flow cytometry histograms (D) and quantification of CD8^+^ T cell proliferation (E) after co‐culture with pre‐treated MDSCs at a ratio of 1:1 (*n* = 3 independent experiments). F) Schematic of BMDM isolation, polarization, treatment, and analysis. G) Relative *Ifnb1* mRNA expression in BMDMs after 6 h of treatment (*n* = 3 independent experiments). H–K) Representative flow cytometry plots (H, J) and quantification (I, K) of macrophage polarization after indicated treatment (*n* = 3 independent experiments). n.s., not significant; ^*^ *p* < 0.05, ^**^ *p* < 0.01, and ^***^ *p* < 0.001.

Myeloid cells, particularly myeloid‐derived suppressor cells (MDSCs) and macrophages, are known to accumulate in bone metastases and contribute to immune suppression and tumor progression [29]. We first examined how factors released from the drug‐loaded GelMA scaffold influence the immunosuppressive functions of MDSCs and bone marrow‐derived macrophages (BMDMs) in vitro. Mouse bone marrow cells were differentiated into MDSCs or BMDMs, and the drug‐loaded GelMA scaffold was incubated in pH 6.5 medium for 48 h to generate a drug‐enriched conditioned medium. This conditioned medium (containing the released MSA‐2 and/or αB7‐H3) was then applied to the cultured MDSCs or BMDMs (Figure 2A,F).

In MDSCs, treatment with the scaffold‐released medium, especially from the dual‐loaded αB7‐H3‐MPs & MSA‐2 scaffold, dramatically reduced the mRNA levels of the immunosuppressive enzymes arginase 1 (*Arg1*) and inducible nitric oxide synthase (*Nos2*) [30], while significantly increasing the expression of interferon beta 1 (*Ifnb1*) (Figure 2B). These changes suggest that the released payloads strongly inhibit the immunosuppressive program of MDSCs. Likewise, BMDMs exposed to MSA‐2‐containing medium showed a pronounced upregulation of *Ifnb1* expression compared to untreated controls (Figure 2G).

To confirm activation of STING signaling, we examined key pathway proteins by Western blot. MDSCs and BMDMs cultured with the scaffold release media for 48 h both exhibited markedly elevated levels of phosphorylated STING (p‐STING) and its downstream phosphorylated interferon regulatory factor 3 (p‐IRF3) (Figure 2C; Figures S4A, S16 and S17). Notably, however, the MSA‐2‐rich media also induced an increase in B7‐H3 protein expression in both MDSCs and BMDMs, consistent with a previous study [16]. Thus, although STING activation by MSA‐2 can trigger robust immune‐stimulatory signaling in myeloid cells, it is accompanied by a concurrent upregulation of the checkpoint molecule B7‐H3, which could contribute to an immunosuppressive microenvironment and potentially limit the efficacy of STING agonist therapy [14, 19].

We next assessed the functional impact of these treatments on T cell suppression. When naive MDSCs were co‐cultured with CD8^+^ T cells, they strongly inhibited T cell proliferation as expected. If MDSCs were pretreated with medium from the MSA‐2‐loaded scaffold, this immunosuppressive effect was partially lifted, allowing more T cells to proliferate. Moreover, if MDSCs were pretreated with the medium from the dual‐loaded αB7‐H3‐MPs & MSA‐2 scaffold, their suppressive effect was almost completely abrogated, leading to a near‐full restoration of CD8^+^ T cell proliferation (Figure 2D,E).

A similar reprogramming was observed in macrophages. Flow cytometry analysis of BMDMs indicated that exposure to the MSA‐2‐containing medium drove a shift from an M2‐like (immunosuppressive) phenotype toward an M1‐like (pro‐inflammatory, tumoricidal) phenotype. This M1 polarization was further enhanced when BMDMs were treated with the dual αB7‐H3‐MPs & MSA‐2 conditioned medium, as evidenced by an increased fraction of cells expressing M1 markers and a reduction in those expressing M2 markers (Figure 2H–K). Thus, the combination of MSA‐2 and αB7‐H3 effectively promotes macrophage repolarization toward a tumor‐suppressive state [31].

Given the central role of dendritic cells (DCs) in antigen presentation and CD8^+^ T‐cell priming, BMDC (bone marrow‐derived dendritic cell) maturation was next assessed in vitro (Figure S2A). Flow cytometry of CD11c^+^ BMDCs showed that MSA‐2‐containing medium increased the frequency of mature CD80^+^CD86^+^ DCs and elevated MHC class II (MHCII) expression compared with medium‐only and blank‐scaffold controls (Figure S2B–E). These results indicate that factors released from the therapeutic scaffold, particularly MSA‐2, can directly promote a DC maturation phenotype consistent with enhanced antigen‐presenting potential [32].

We also evaluated STING pathway activation in tumor cells under these conditions using the Myc‐Cap prostate cancer cell line that preferentially metastasizes to bone [33], as our model system (Figure S3A). Myc‐Cap tumor cells treated with MSA‐2‐enriched scaffold medium exhibited a significant increase in *Ifnb1* gene expression (Figure S3B). Immunofluorescence staining showed that MSA‐2 treatment led to strong nuclear localization of p‐IRF3 in Myc‐Cap cells (Figure S3C), indicating active STING pathway signaling. Consistently, Western blotting confirmed elevated levels of p‐STING and p‐IRF3 in Myc‐Cap cells after exposure to MSA‐2 (Figures S4B and S18). Notably, MSA‐2 treatment also upregulated B7‐H3 protein in these tumor cells, mirroring the effect seen in myeloid cells.

Taken together, STING pathway activation by MSA‐2 in MDSCs, macrophages, and tumor cells induces a potent type I interferon response (e.g., IFNβ) but also undesirably elevates B7‐H3 expression, which could dampen the immune response. These findings provide a clear rationale for combining the STING agonist with B7‐H3 checkpoint blockade: the former converts the tumor milieu from “cold” to “hot”, and the latter prevents the emerging B7‐H3‐mediated immunosuppression, thereby maximizing antitumor immunity.

### 2.3 In Vitro Immune Response Activation and Antitumor Efficacy of the Dual‐Drug Scaffold

**FIGURE 3:**
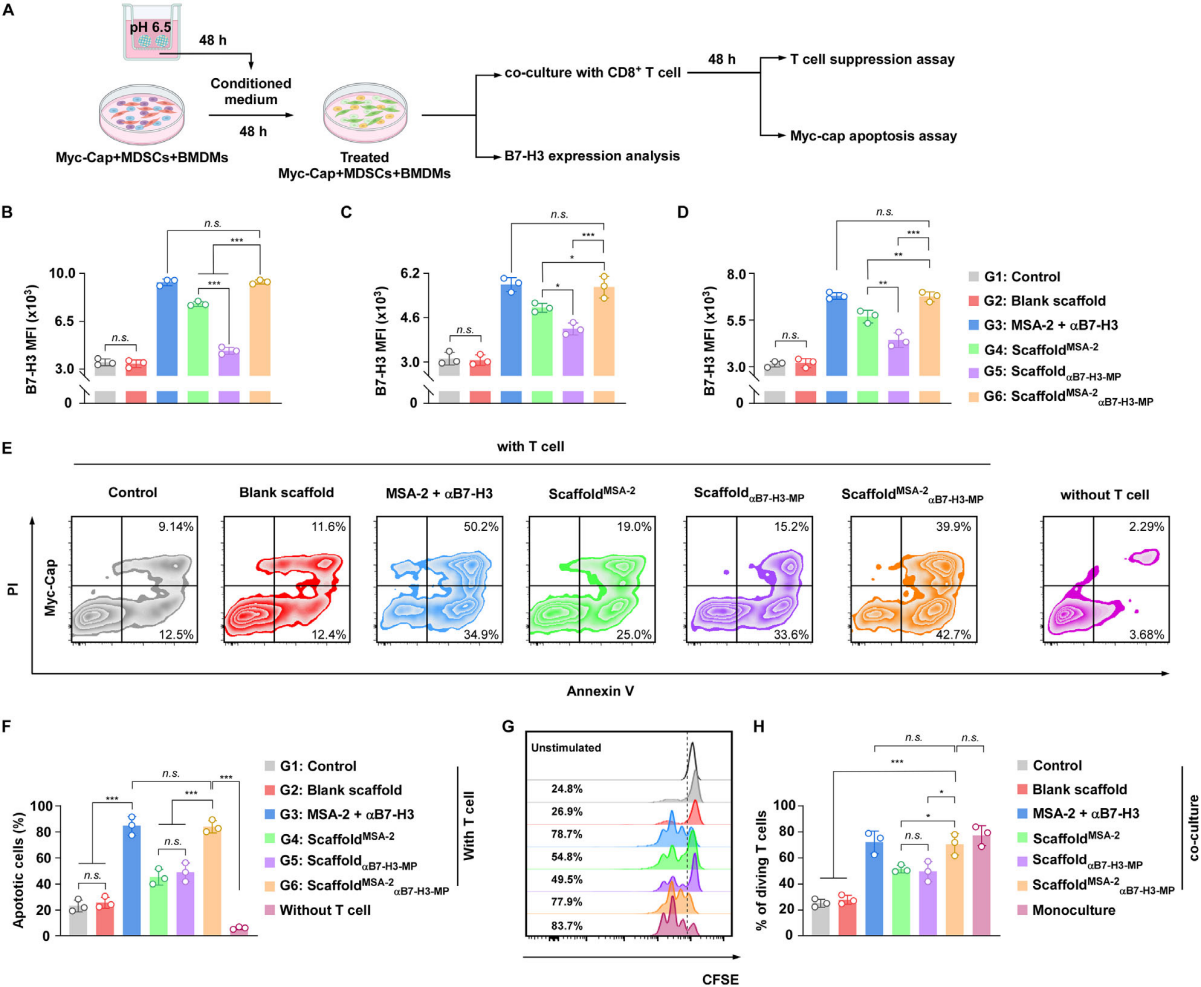
In vitro activation of immune responses. A) Workflow schematic for co‐culture of Myc‐Cap, MDSCs, and BMDMs, followed by analysis of surface B7‐H3 expression, CD8^+^ T cell proliferation, and Myc‐Cap apoptosis. B–D) Quantification of surface B7‐H3 expression in Myc‐Cap (B), MDSC (C), and BMDM (D) cells after 48 h of treatment (*n* = 3 independent experiments). E, F) Representative apoptosis plots (E) and quantitative analysis (F) of apoptosis in Myc‐Cap cells post‐treatment (*n* = 3 independent experiments). G, H) Representative CFSE dilution plots (G) and proliferation quantification (H) of CD8^+^ T cell co‐cultured with pre‐treated Myc‐Cap, MDSCs, and BMDMs at a ratio of 3:1:1:1 (*n* = 3 independent experiments). n.s., not significant; ^*^ *p* < 0.05, ^**^ *p* < 0.01, and ^***^ *p* < 0.001.

To further evaluate how combining MSA‐2 with B7‐H3 blockade enhances antitumor immunity, we established a co‐culture system mimicking the tumor microenvironment. MDSCs, BMDMs, and Myc‐Cap tumor cells were co‐cultured together for 48 h in the presence of the scaffold‐released medium (containing either MSA‐2 alone or MSA‐2 plus αB7‐H3), as outlined in Figure 3A. After this preconditioning, we analyzed B7‐H3 surface expression on each cell type. We then introduced CFSE‐labeled CD8^+^ T cells to the co‐cultures and incubated for another 48 h, after which CD8^+^ T cell proliferation and tumor cell apoptosis were measured (Figure 3A).

Consistent with our earlier observations, exposure to MSA‐2‐laden medium led to a significant upregulation of surface B7‐H3 on all three cell types (MDSCs, BMDMs, and Myc‐Cap tumor cells) (Figure 3B–D; Figure S5). Importantly, the MSA‐2 pre‐treatment rendered the tumor cells more susceptible to T cell‐mediated killing. When CD8^+^ T cells were added, the MSA‐2‐conditioned tumor cells underwent significantly higher rates of apoptosis compared to untreated controls (Figure 3E,F). B7‐H3 signaling is known to inhibit T cell and NK cell effector functions. For example, it can suppress the secretion of crucial effector cytokines like IFNγ and TNFα by T cells [34]. Additionally, it also diminishes the immune responses of natural killer (NK) cells and T cells, thereby facilitating tumor immune evasion [34, 35, 36]. Consistent with this, when the αB7‐H3 antibody was also present (i.e., in the dual‐treatment medium), we observed an even greater degree of tumor cell apoptosis (Figure 3E,F). Thus, STING pathway activation by MSA‐2 primes tumor cells for immune‐mediated destruction, and concurrent B7‐H3 checkpoint blockade amplifies this antitumor effect.

We also evaluated CD8^+^ T cell proliferation in these co‐cultures by tracking CFSE dilution. In co‐cultures without any treatment, proliferating CD8^+^ T cells are scarce due to suppressive factors from MDSCs, BMDMs, and tumor cells (Figure 3G,H). MDSC/BMDM/tumor cell cultures pretreated with MSA‐2‐containing medium showed a partial restoration of T cell proliferation, evidenced by increased CFSE dilution. Notably, when the co‐culture was pretreated with medium from the scaffold^MSA‐2^_αB7‐H3‐MP_, the suppression of T cell proliferation was almost completely reversed, and CD8^+^ T cells proliferated robustly.

In short, combining MSA‐2 with B7‐H3 blockade in vitro effectively counteracts the immunosuppressive influence of MDSCs and tumor cells. The dual‐treatment significantly enhances CD8^+^ T cell proliferation and cytotoxic function, leading to greater tumor cell apoptosis than either treatment alone. These results underscore the synergistic potential of simultaneous STING activation and B7‐H3 checkpoint inhibition in provoking a strong antitumor immune response.

### 2.4 Immunological Response against Tumors In Vivo following the Drug‐Loaded GelMA Scaffold Implantation

**FIGURE 4:**
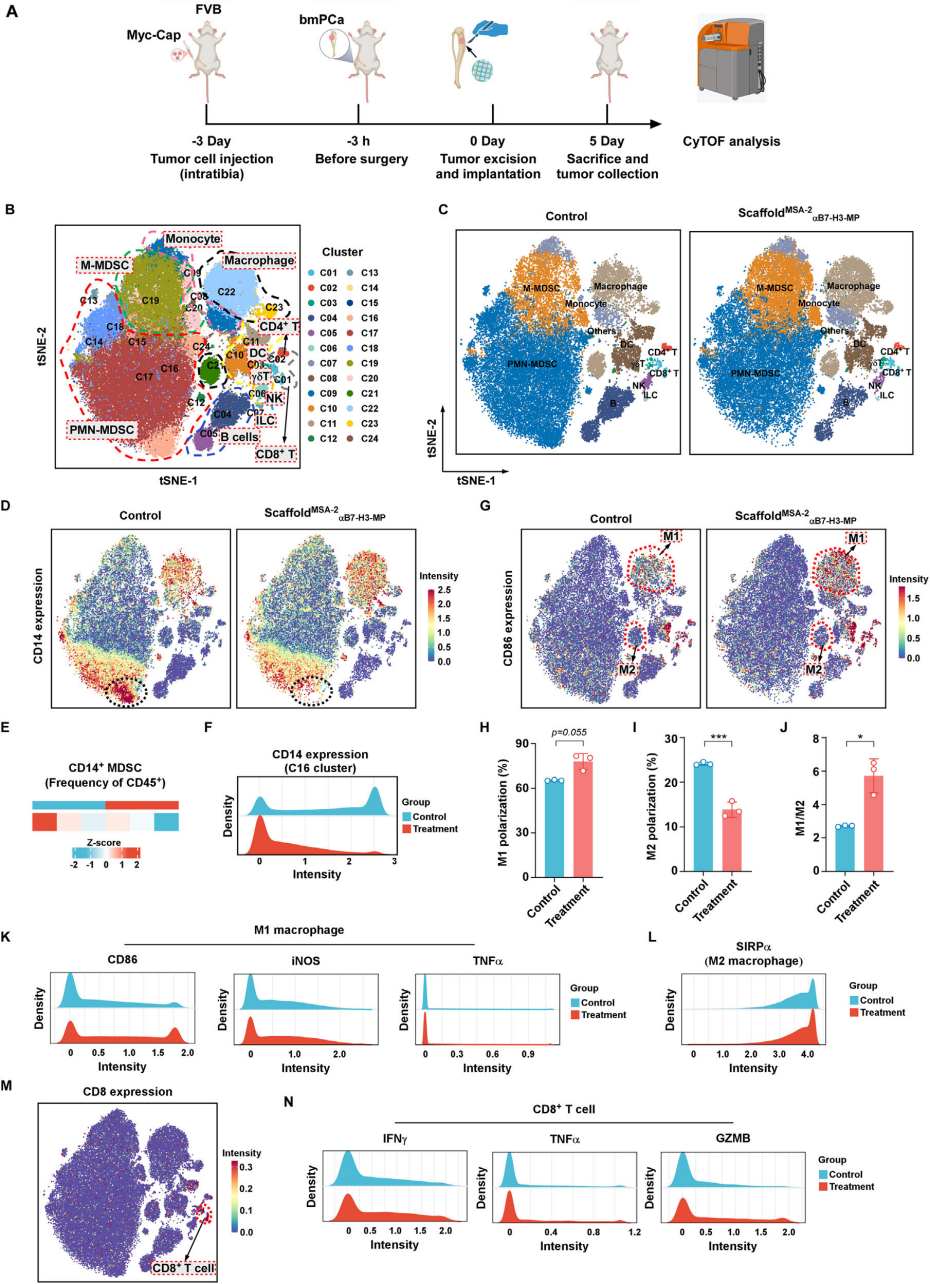
CyTOF analysis of antitumor immune response in bmPCa after scaffold implantation. A) Schematic diagram displaying the animal experimental procedures, including establishment of Myc‐Cap prostate cancer bone metastasis model via intratibial tumor cell injection, tumor resection, scaffold implantation, sample harvest, and CyTOF analysis on day 5 post‐implantation. B) The t‐SNE plot depicting the distribution of immune cells from tumor‐bearing tibiae across all groups. C) The t‐SNE plot comparing the distribution of immune cells in the control group and scaffold^MSA‐2^_αB7‐H3‐MP_ group by CyTOF analysis. D) t‐SNE diagram illustrating the difference between two groups of MDSCs based on the functional marker CD14. The black circle indicates the cell population with high expression of the CD14 protein. E) Heatmap showing the frequency of the CD14^+^ MDSC subset. F) Differential expression density of CD14 protein within the CD14^+^ MDSC subset. G) t‐SNE diagram depicting the difference between two groups in terms of M1 macrophage marker CD86. The red circle indicates the cell population of M1 or M2 macrophages. H–J) Quantified proportions of M1 (H), M2 (I), and M1/M2 ratio (J) in intratumor macrophages (*n* = 3 per group). K, L) Differential expression of CD86, iNOS, and TNFα in M1 macrophages (K), and SIRPα in M2 macrophages (L). M) t‐SNE diagram displaying expression patterns of CD8. The red circle indicates the CD8^+^ T cell population. N) Differential expression density of IFNγ, TNFα, and GzmB in CD8^+^ T cells. ^*^ *p* < 0.05, and ^***^ *p* < 0.001.

Encouraged by the in vitro findings, we next investigated whether the scaffold^MSA‐2^_αB7‐H3‐MP_ could provoke a robust antitumor immune response in vivo. We established a bone metastatic prostate cancer (bmPCa) model by injecting Myc‐Cap cells into the tibia of FVB mice. Three days later, we performed an incomplete surgical resection of the tumor (to mimic residual disease after surgery) and implanted a scaffold into the bone defect (Figure 4A; Figure S6). Mice were randomized to receive either the dual‐loaded scaffold (scaffold^MSA‐2^_αB7‐H3‐MP_) or no scaffold (control). After 5 days, the treated and control tumor‐bearing tibiae were harvested for immune profiling by mass cytometry (CyTOF).

Immune cells were isolated from the tumor‐bearing tibiae and stained with a panel of surface and intracellular markers for CyTOF analysis (Figure S7A). t‐Distributed stochastic neighbor embedding (t‐SNE) visualization of the data revealed 24 distinct immune cell subpopulations, including MDSCs, CD8^+^ T cells, CD4^+^ T cells, macrophages, B cells, monocytes, dendritic cells (DCs), natural killer (NK) cells, gamma‐delta T (γδT) cells, and others (Figure 4B,C; Table S2). We compared the immune composition between scaffold‐treated and control mice. Overall, the immune cell landscape was broadly similar, with the exception of a notable increase in monocytes in the treated group (Figure S7B).

Despite the general similarity in major immune populations, we observed a significant reduction in a specific MDSC subset in the treated mice. In particular, the frequency of cluster 16 (defined as CD14^+^ MDSC) was lower in the scaffold‐treated group than in controls (Figure 4D,E). Moreover, the expression level of CD14 within this cluster was also diminished after treatment (Figure 4F; Figure S7C). CD14 is a marker associated with immune‐suppressive polymorphonuclear myeloid‐derived suppressor cells (PMN‐MDSCs) [37], which can inhibit T cell activity and promote tumor progression. The decrease of this CD14^+^ MDSC population suggests that the scaffold is mitigating a key immunosuppressive component of the tumor microenvironment.

Macrophage populations in the bone marrow were also markedly altered by the treatment. The scaffold‐treated group showed a higher prevalence of M1 macrophages (cluster 22) and a lower prevalence of M2 macrophages (cluster 21) compared to controls, resulting in a significantly increased M1/M2 ratio (Figure 4G–J). Consistently, M1‐associated markers such as CD86, iNOS, and TNFα were upregulated in the treated tumors (Figure S7D–F), indicative of a pro‐inflammatory, antitumor macrophage phenotype. Furthermore, we noted that the expression of SIRPα, an inhibitory receptor on monocytes/macrophages that binds CD47 to suppress phagocytosis [38], was substantially reduced on the remaining M2 macrophages in treated mice (Figure 4L; Figure S7G). Collectively, these data demonstrate that the scaffold therapy skews the myeloid compartment toward an immune‐stimulating phenotype, reducing immunosuppressive MDSCs and M2 macrophages while enriching for macrophages with M1 characteristics.

Concomitant with these myeloid changes, we observed enhancements in the T cell compartment. CD8^+^ T cells in treated mice exhibited higher expression of cytotoxic effector molecules, including IFNγ, TNFα, and GzmB, relative to the control group (Figure 4M,N; Figure S7H−J). Accordingly, the proportion of GzmB^+^CD8^+^ T cells and IFNγ^+^CD8^+^ T cells was increased in the treatment group (Figure S7K–N), indicating an expansion of highly cytotoxic T cells in the tumor microenvironment.

Other immune subsets showed similar trends toward heightened antitumor activity following scaffold treatment. Tumor‐infiltrating NK cells from treated mice tended to express higher levels of GzmB, TNFα, and IFNγ (Figure S8A–D), suggesting increased NK cell activation. Dendritic cells in the treated group upregulated the co‐stimulatory maturation marker CD86 (Figure S8E–G), consistent with enhanced antigen‐presenting capability. Meanwhile, the population of regulatory T cells (Treg) was notably reduced with treatment (Figure S8H,I), relieving another source of immunosuppression. Thus, the scaffold rapidly reshapes the bone tumor microenvironment from an immune‐suppressive state toward an immune‐activated state.

In sum, just five days after implantation, the scaffold^MSA‐2^_αB7‐H3‐MP_ induced broad immunological changes in the bone metastatic niche: suppressive myeloid cells were diminished or repolarized, while cytotoxic lymphocytes and other effector cells were invigorated. These early immunomodulatory effects provide a strong foundation for subsequent antitumor activity and validate the strategy of converting a “cold” bone metastasis into a “hot” immunologically active tumor site.

### 2.5 Therapeutic Efficacy of the Scaffold in Preventing Postoperative Tumor Recurrence

**FIGURE 5:**
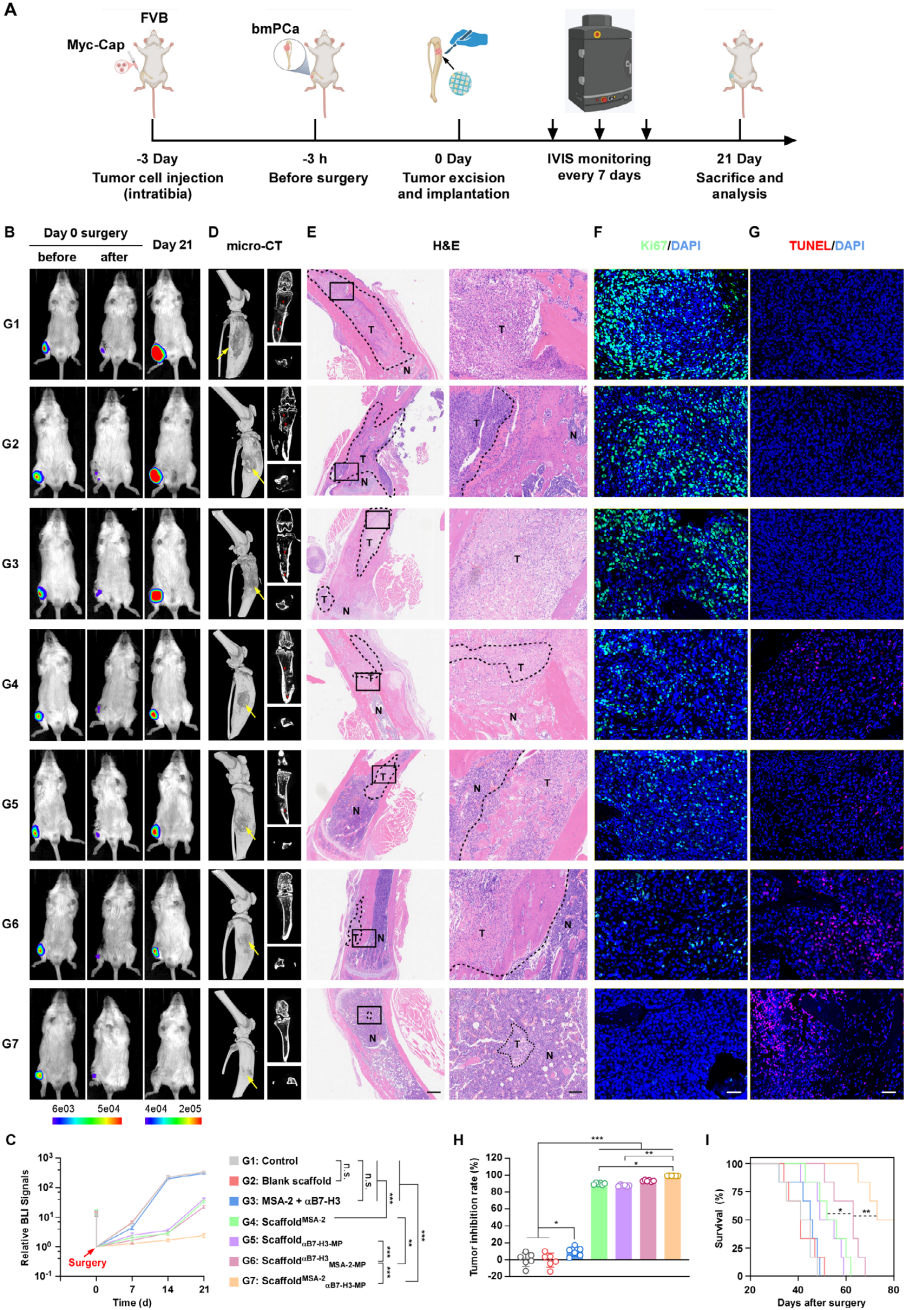
In vivo antitumor efficacy of the scaffold^MSA‐2^_αB7‐H3‐MP_ in bmPCa. A) Schematic diagram displaying the animal experimental workflow. B) Representative BLI images of tumor progression in tumor‐bearing FVB mice before surgery, immediately after surgery, and 21 days after treatment across all experimental groups. C) Quantified BLI signals in tumor‐bearing tibiae of mice from the indicated groups (*n* = 6 per group). D) Representative micro‐CT 3D reconstructions and sectional images of intratibial lesions in murine models at 21 days post‐treatment (*n* = 6 per group; yellow arrows indicate surgery sites, and red arrows indicate osteoblastic lesions). E) Representative H&E‐stained tibial sections from the specified groups (T, tumor; N, adjacent tissue). Scale bars, 500 µm (left) and 100 µm (right). F) Representative immunofluorescence staining images of Ki67 in tumor sections from different groups (*n* = 3 mice). Scale bar, 50 µm. G) Representative TUNEL fluorescence images of tumor‐bearing tibiae from indicated groups (*n* = 3 mice). Scale bar, 50 µm. H) Tumor inhibition rate calculated based on BLI signal intensities in intratibial tumors from indicated groups (*n* = 6 per group). I) Survival curves of mice from different groups (*n* = 6 per group). n.s., not significant; ^*^ *p* < 0.05, ^**^ *p* < 0.01, and ^***^ *p* < 0.001.

Using the bone metastasis model described above, we evaluated the scaffold's ability to prevent tumor recurrence after surgical resection (Figure 5A). After resecting the primary tumor mass (achieving at least 90% tumor debulking as confirmed by a sharp drop in bioluminescence signal; Figure 5B,C), mice were assigned to one of seven treatment groups: G1 (control group, surgery only, no additional therapy), G2 (Blank scaffold, surgery + implantation of a drug‐free scaffold), G3 (MSA‐2 + αB7‐H3, surgery + intratibial injection of free MSA‐2 and αB7‐H3), G4 (scaffold^MSA‐2^, surgery + implantation of a MSA‐2‐loaded scaffold), G5 (scaffold_αB7‐H3‐MP_, surgery + implantation of a scaffold containing CaCO_3_ microparticles pre‐loaded with the anti‐B7‐H3 antibody), G6 (scaffold ^αB7‐H3^_MSA‐2‐MP_, surgery + implantation of a scaffold containing MSA‐2 encapsulated in CaCO_3_ microparticles and free αB7‐H3 mixed in the scaffold, the reverse release sequence of the main scaffold), and G7 (scaffold^MSA‐2^_αB7‐H3‐MP_, surgery + implantation of the dual‐loaded scaffold with B7‐H3 antibody encapsulated in CaCO_3_ microparticles and free MSA‐2 mixed in the scaffold, sequential release design). Tumor growth in the tibia was monitored weekly by bioluminescence imaging (BLI) for 21 days. Tumor recurrence and progression were quantified by calculating tumor growth inhibition rates from the BLI data (see Experimental Section for details).

The BLI signal curves of the bmPCa are shown in Figure 5C and Figure S9A–G. As expected, both the control group (G1) and blank scaffold group (G2) exhibited a rapid resurgence of tumor signal, indicating aggressive recurrence in the absence of effective therapy. In contrast, all groups that received therapeutic agents (G3–G7) demonstrated significantly slower tumor growth. Notably, the mice that received a one‐time injection of free MSA‐2 + αB7‐H3 (G3) exhibited only a modest delay in tumor regrowth compared to controls, suggesting that a single bolus of unencapsulated drugs could not maintain sufficient local concentrations in the tumor bed, likely due to the rapid drug diffusion and clearance [24].

Importantly, the manner in which the drugs were delivered had a profound impact on efficacy. The dual‐drug reservoir system with sequential delivery (G7: scaffold^MSA‐2^_αB7‐H3‐MP_) outperformed all other treatments, including the reverse‐sequence scaffold (G6: scaffold ^αB7‐H3^_MSA‐2‐MP_). By releasing MSA‐2 first and αB7‐H3 subsequently, the G7 scaffold achieved nearly complete tumor growth inhibition. Quantitatively, the G7 group exhibited a ∼100% tumor inhibition rate by study end, whereas the G3 (free drug, 9.5%) and G6 (reverse sequence, 93.3%) groups showed considerably lower inhibition (Figure 5H). Correspondingly, all mice treated with the scaffold^MSA‐2^_αB7‐H3‐MP_ (G7) survived throughout the 60 day observation period, with a survival rate significantly higher than that of the other groups (Figure 5I).

To correlate these findings with structural outcomes in bone, we performed micro–computed tomography (Micro‐CT) on the excised tumor‐bearing tibiae from each group. (Figure 5D). 3D reconstruction and cross‐sectional images revealed severe cancer‐induced bone destruction in the control (G1), blank scaffold (G2), and free drug (G3) groups, evident as extensive osteolytic lesions. By contrast, tibiae from the sequential dual‐drug scaffold group (G7) maintained much more normal bone architecture, suggesting that tumor growth was effectively controlled and bone integrity preserved. Because the Myc‐Cap model exhibits mixed osteoblastic and osteolytic activity [39, 40], the bone volume to tissue volume ratio (BV/TV) did not differ significantly among groups (Figure S9H), likely reflecting the combined effects of tumor removal, bone remodeling, or tumor recurrence in mice. However, the bone surface area to bone volume ratio (BS/BV) was markedly lower in the G7 scaffold group (Figure S9I), indicating smoother, less eroded bone surfaces consistent with reduced tumor burden and active bone regeneration. Thus, the successful local immunotherapy not only inhibited tumor recurrence but also mitigated cancer‐related bone damage.

Histological analysis further corroborated these outcomes. Hematoxylin and eosin (H&E) staining of tibiae sections showed that the G7 scaffold‐treated mice had only microscopic remnants of tumor, whereas other groups harbored large residual tumor masses (Figure 5E). Immunofluorescent staining for Ki67, a marker of cell proliferation, revealed very few proliferating (Ki67^+^, green) tumor cells in the G7 group, in stark contrast to the high number of Ki67^+^ cells in control and other treatment groups (Figure 5F; Figure S9J). Additionally, TUNEL staining for apoptotic cells showed extensive cell death in the G7 scaffold tumors, with the highest proportion of TUNEL^+^ (red, indicating apoptosis) cells among all groups (Figure 5G; Figure S9K). These findings confirm that the scaffold^MSA‐2^_αB7‐H3‐MP_ effectively suppresses tumor cell proliferation and induces tumor cell apoptosis in vivo, aligning with our in vitro results.

Finally, we assessed the systemic safety of our localized treatment approach. Blood chemistry analysis indicated that liver enzymes (ALT and AST) and renal function markers (UREA and CREA) remained within the normal ranges in all treated mice (Figure S10A–D), with no significant differences from untreated controls. Additionally, H&E examinations of major organs, including heart, liver, spleen, lung, and kidney, revealed no histopathological abnormalities or overt inflammatory damage attributable to the treatments (Figure S10E). These results suggest that in situ delivery of MSA‐2 and αB7‐H3 via the GelMA scaffold did not produce systemic toxicity, underscoring the safety of this local immunotherapy strategy.

### 2.6 Remodeling of the Tumor Microenvironment by Scaffold Therapy

To assess the durability of the immune reprogramming induced by the scaffold, we analyzed immune cell populations in the bone tumor microenvironment 21 days after treatment using flow cytometry. The gating strategies for identifying MDSCs, macrophages, and T cells are shown in Figure S11.

**FIGURE 6:**
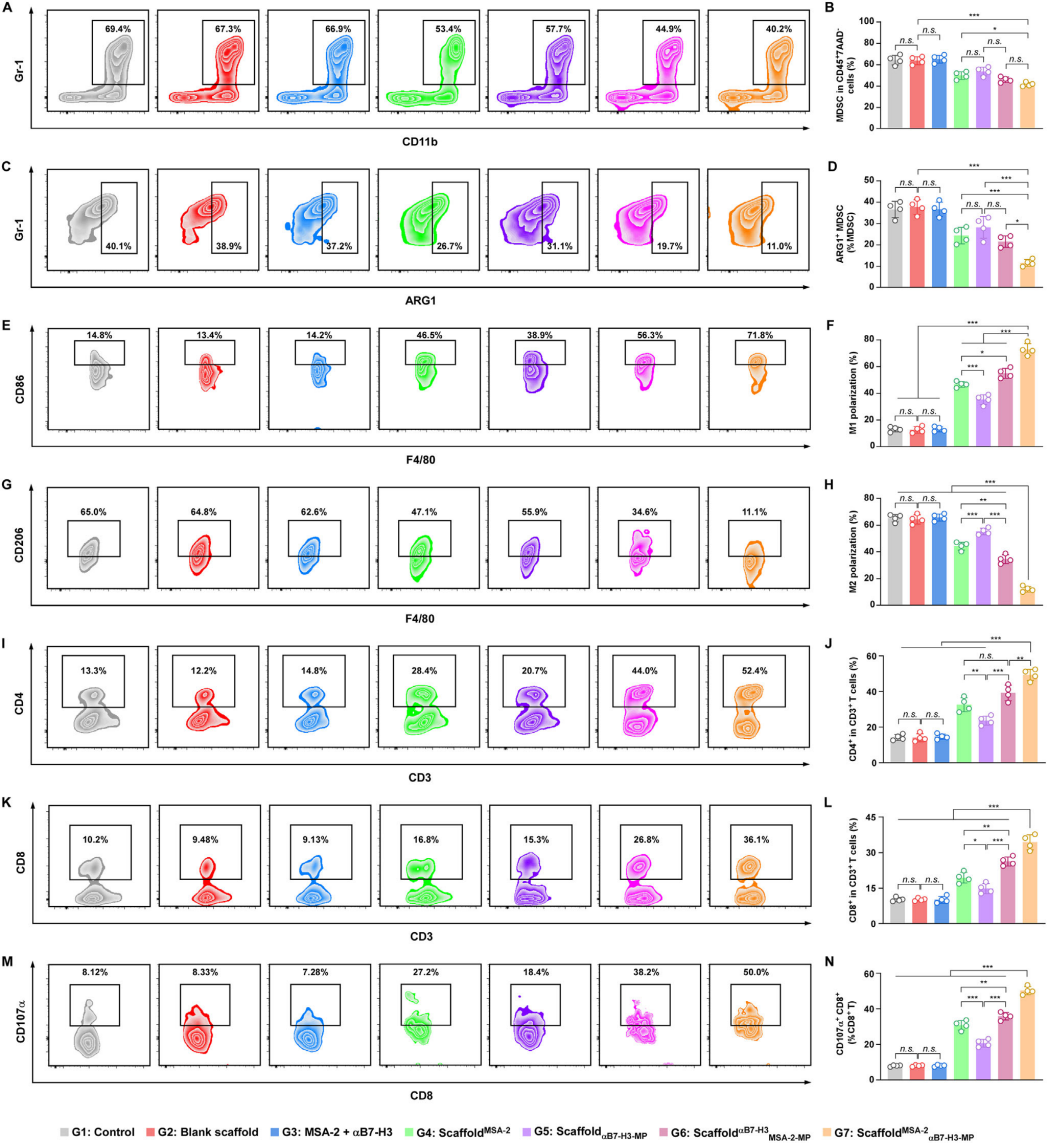
Remodeling of the TME by the scaffold^MSA‐2^_αB7‐H3‐MP_. Representative flow cytometry plots and quantified percentages of MDSCs (A, B), ARG1^+^ MDSCs (C, D), M1 macrophages (E, F), M2 macrophages (G, H), CD4^+^ T cells (I, J), CD8^+^ T cells (K, L), and CD107α^+^CD8^+^ T cells (M, N) in the TME following different treatments (*n* = 4 per group). n.s., not significant; ^*^ *p* < 0.05, ^**^ *p* < 0.01, and ^***^ *p* < 0.001.

The scaffold^MSA‐2^_αB7‐H3‐MP_ treatment resulted in a markedly lower accumulation of immunosuppressive myeloid cells compared to other treatments. The proportion of total MDSCs was reduced, and within the MDSC compartment, the fraction of arginase‐1‐expressing MDSCs (ARG1^+^ MDSCs) was significantly lower in the scaffold‐treated group (Figure 6A–D). Similarly, the scaffold‐treated tumors contained fewer M2‐polarized macrophages (Figure 6G,H) and a higher proportion of M1 macrophages relative to controls (Figure 6E,F). These data confirm that the local dual‐drug therapy leads to a sustained polarization of the bone marrow tumor microenvironment away from an immunosuppressive phenotype and toward a more pro‐inflammatory state.

Correspondingly, the scaffold therapy greatly enhanced T cell presence and activation in the tumors. Both CD4^+^ T cells and CD8^+^ T cells were significantly more abundant in the scaffold^MSA‐2^_αB7‐H3‐MP_ group compared to controls (Figure 6I–L). Importantly, the frequency of cytotoxic CD8^+^ T cells, as evidenced by CD107α expression (a marker of T cell degranulation), was dramatically higher in the scaffold‐treated tumors (Figure 6M,N). This indicates that not only are more T cells present, but a larger fraction of them are functional cytotoxic effectors actively engaging the tumor.

The significance of the sequential release strategy was further underscored by comparing the dual‐drug scaffold (G7) with the reverse‐sequence version (G6). Mice treated with the G6 displayed higher levels of MDSCs (including ARG1^+^ MDSCs) and more M2 macrophages, alongside reduced frequencies of CD4^+^ T cells, CD8^+^ T cells, and CD107α^+^CD8^+^ cytotoxic T cells, relative to the G7 sequential‐release group. Thus, only the correctly sequenced delivery (MSA‐2 first, then αB7‐H3) produced the full beneficial reprogramming of the tumor immune microenvironment, highlighting the synergistic requirement of STING activation followed by checkpoint blockade for maximal antitumor immunity.

### 2.7 Evaluation of Long‐Term Immune Memory against Tumors

**FIGURE 7:**
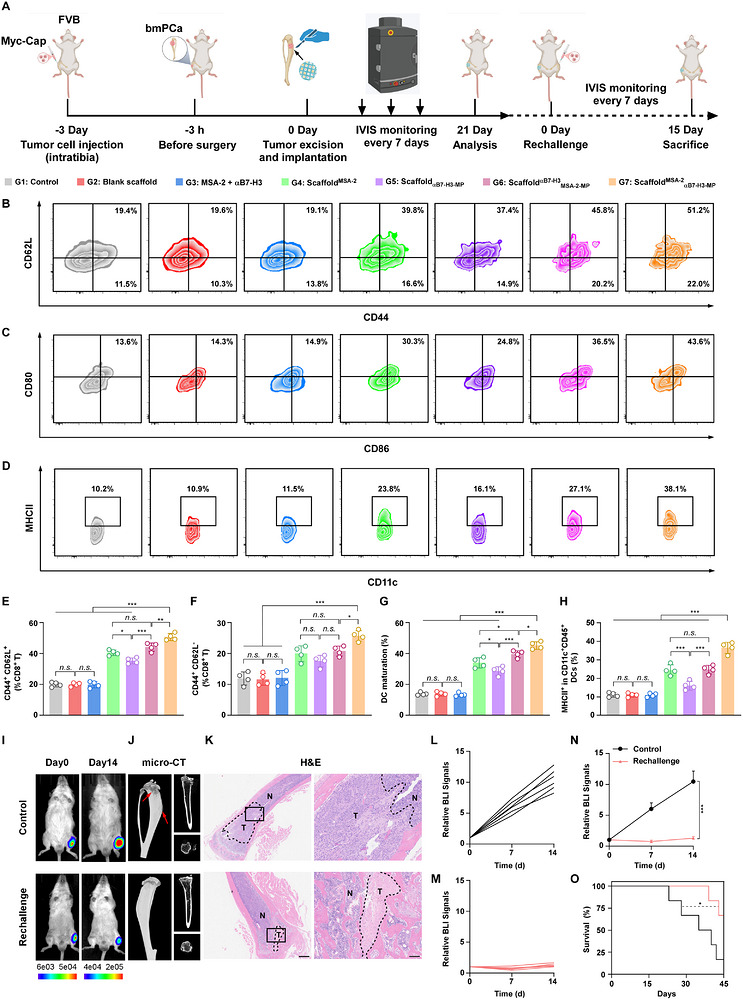
Induction of durable antitumor immunity by scaffold treatment. A) Experimental design for tumor rechallenge with contralateral Myc‐Cap cells intratibial injection. B–H) Representative flow cytometry plots (B–D) and quantification (E–H) of T_CM_ (CD44^+^CD62L^+^) (B, E), T_EM_ (CD44^+^CD62L^–^) (B, F) cells, matured DCs (C, G), and MHCII^+^ DCs (D, H) in splenic cells of mice upon different treatments (*n* = 4 per group). I) Representative BLI images of rechallenged tumors immediately and 14 days post‐rechallenge. J) Representative micro‐CT 3D reconstruction and sectional images of tumor‐bearing tibiae from mice on day 14 post‐rechallenge (red arrows indicate osteolytic lesions, *n* = 6 per group). K) Representative H&E‐stained sections of the tibia from the indicated groups (T, tumor; N, adjacent tissue). Scale bars, 500 µm (left) and 100 µm (right). L–N) Individual (L, M) and mean (N) BLI signal intensities of tumor‐bearing tibiae from indicated groups (*n* = 6 per group). O) Survival curves of rechallenged mice monitored over 45 days post‐treatment (*n* = 6 per group). n.s., not significant; ^*^ *p* < 0.05, ^**^ *p* < 0.01, and ^***^ *p* < 0.001.

Immunological memory, orchestrated by adaptive immunity, plays a crucial role in preventing tumor recurrence and metastasis [41]. Therefore, we investigated whether the local scaffold therapy could induce long‐lasting antitumor immunity. To this end, mice that achieved complete tumor regression after scaffold^MSA‐2^_αB7‐H3‐MP_ treatment were subjected to a tumor rechallenge. These mice were injected with Myc‐Cap tumor cells in the tibia of the opposite (previously untreated) leg (Figure 7A). As a control, age‐matched naive mice received the same tibial injection of Myc‐Cap cells. Tumor growth in the rechallenged and control mice was then monitored by BLI over the subsequent 14 days.

To investigate the immune correlates of the long‐term protection, we analyzed splenocytes from the rechallenged mice for T cell memory phenotypes on day 21 (Figure S13). Mice cured by the scaffold^MSA‐2^_αB7‐H3‐MP_ displayed significantly higher frequencies of CD44^+^CD62L^−^ effector memory T (T_EM_) cells and CD44^+^CD62L^+^ central memory T (T_CM_) cells among their T cell population (Figure 7B,E–F). In contrast, control mice had far fewer memory T cells. This enrichment of memory T cells in treated mice suggests that the scaffold therapy generated a pool of tumor‐specific memory lymphocytes capable of mounting a rapid response upon tumor re‐exposure.

We also examined the activation status of intrasplenic DCs (Figure 7C,D), as DCs are crucial for priming and maintaining T cell responses. Spleens from scaffold^MSA‐2^_αB7‐H3‐MP_‐treated mice contained a higher proportion of mature DCs (CD80^+^CD86^+^) compared to other groups (Figure 7G), indicating an activated phenotype. Moreover, the frequency of MHC‐class‐II positive (MHCII^+^) DCs was elevated in the treated mice (Figure 7H), suggesting enhanced antigen presentation capacity.

The scaffold^MSA‐2^_αB7‐H3‐MP_‐treated mice exhibited robust resistance to tumor rechallenge. By day 14 after rechallenge, the bioluminescence signal of tumors in the pre‐treated group was nearly an order of magnitude lower than that in control mice (Figure 7I,L–N). Micro‐CT analysis corroborated this result: the rechallenged tibiae in previously treated mice showed significantly less bone destruction (higher BV/TV and lower BS/BV) compared to the tibiae of control mice (Figure 7J; Figure S12). Consistent with the Micro‐CT results, H&E staining revealed much smaller tumor lesions in the rechallenge group vs the control group (Figure 7K). All mice in the pre‐treated (scaffold) group survived the rechallenge period, whereas the control mice showed rapid mortality, with survival dropping to nearly 15% by the end of observation (Figure 7O). These results indicate that the dual‐drug scaffold therapy induced a durable protective immunity that can prevent tumor recurrence after surgical resection, and the systemic immune changes (augmented memory T cells and activated DCs) likely underpin the observed long‐term tumor immunity in the rechallenged animals.

Consistent with earlier findings, mice that received the reverse‐sequence scaffold (scaffold ^αB7‐H3^_MSA‐2‐MP_) exhibited weaker long‐term immune responses. They had fewer T_EM_ and T_CM_ cells, as well as a lower fraction of activated splenic DCs, compared to the sequential‐release scaffold group. This further emphasizes that the proper timing of STING agonist and checkpoint antibody release is critical for achieving not only acute tumor control but also durable antitumor immunity.

### 2.8 Efficacy of the scaffold in multiple bone metastasis models

**FIGURE 8:**
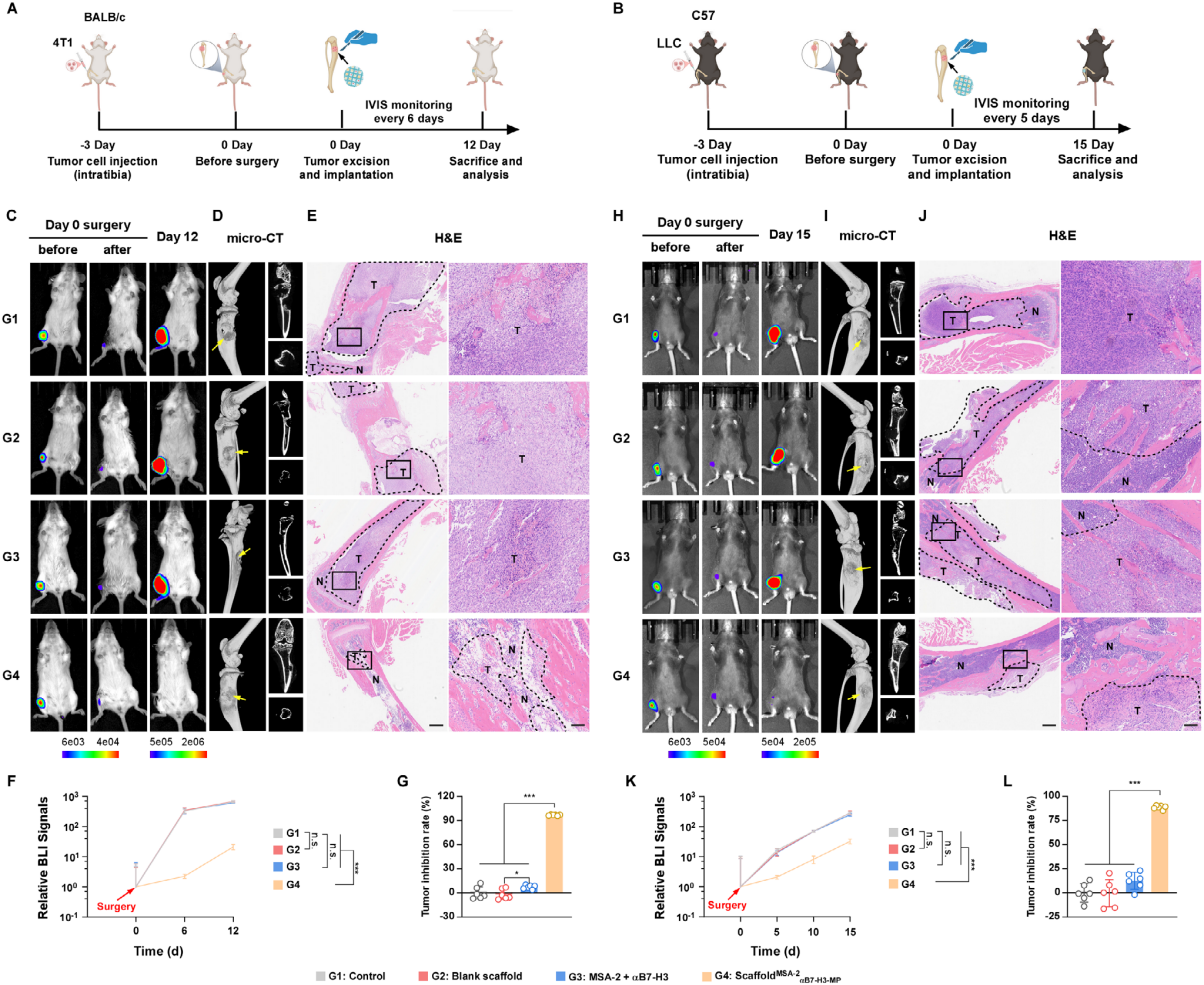
Antitumor effects of scaffold treatment in bone metastatic 4T1 and LLC tumor models. A, B) Schematic illustration depicting the establishment of a bone metastatic breast cancer model via intratibial injection of 4T1 cells into BALB/c mice (A) and a bone metastatic lung cancer model through intratibial injection of LLC cells into C57BL/6 mice (B). C, H) Representative BLI images of tumor‐bearing BALB/c mice (C) and C57BL/6 mice (H) before surgery, immediately after surgery, and at indicated time points. D, I) Representative micro‐CT 3D reconstruction and sectional images of tumor‐bearing tibiae from each group (yellow arrows indicate surgery sites, *n* = 6 per group). E, J) Representative H&E‐stained tibial sections from the specified groups (T, tumor; N, adjacent tissue). Scale bars, 500 µm (left) and 100 µm (right). F, K) Quantified BLI signals in intratibial 4T1 (F) and LLC (K) tumors of mice from the indicated groups (n = 6 per group). G, L) Tumor inhibition rates calculated based on BLI signal intensities in intratibial 4T1 (G) and LLC (L) tumors from indicated groups (*n* = 6 per group). n.s., not significant; ^*^ *p* < 0.05, and ^***^ *p* < 0.001.

To demonstrate the generalizability of our approach, we tested the scaffold^MSA‐2^_αB7‐H3‐MP_ in additional bone metastasis models. We selected a 4T1 murine breast cancer model and an LLC (Lewis lung carcinoma) murine lung cancer model, both of which are known to highly express B7‐H3 [42, 43]. In each case, tumor cells were injected into the tibia to establish bone lesions, and three days later, a tumor debulking surgery was performed to remove ∼90% of the tumor mass. The mice were then treated with either G1 (control, no scaffold), G2 (blank scaffold), G3 (free MSA‐2 + αB7‐H3), or G4 (scaffold^MSA‐2^_αB7‐H3‐MP_). Tumor progression was monitored by BLI, and at the end of the study, tibiae were collected for Micro‐CT and H&E histological analysis (Figure 8A,B).

In the 4T1 bone metastasis model, the dual‐drug scaffold again produced superior therapeutic outcomes (Figure 8A). Mice treated with the scaffold^MSA‐2^_αB7‐H3‐MP_ had dramatically lower BLI signals in the tibia over time compared to all other groups (Figure 8C,F; Figure S14A–D), reflecting a strong suppression of tumor recurrence. Accordingly, the scaffold group exhibited the highest tumor growth inhibition rate among the groups (Figure 8G). Micro‐CT scans of the tibiae showed that scaffold‐treated mice retained significantly more bone volume (higher BV/TV ratio) and had smoother bone surfaces (lower BS/BV ratio) relative to controls (Figure 8D; Figure S14E,F), indicating that tumor‐induced bone destruction was significantly suppressed, and bone remodeling at the surgical defect site was occurring. Additionally, H&E staining revealed only a small residual tumor lesion in the scaffold^MSA‐2^_αB7‐H3‐MP_‐treated bones, whereas much larger tumors were present in control and free‐drug‐treated mice (Figure 8E).

A similar pattern was observed in the LLC lung cancer bone metastasis model (Figure 8B). Scaffold^MSA‐2^_αB7‐H3‐MP_‐treated mice showed minimal tumor recurrence and progression by BLI, whereas control and blank groups had rapidly increasing signals (Figure 8H,K,L; Figure S15A–D). The scaffold^MSA‐2^_αB7‐H3‐MP_ significantly inhibited tumor progression, leading to high tumor suppression rates (Figure 8L). Micro‐CT analysis demonstrated that the scaffold protected the bone from destruction, as evidenced by preservation of bone structure in treated tibiae compared to the extensive osteolytic damage seen in controls (Figure 8I; Figure S15E,F). Moreover, histological examination showed only minor tumor involvement in scaffold‐treated bones, whereas large tumors filled the bone marrow cavity in untreated mice (Figure 8J). These results confirm that the potent antitumor immune effects of the dual‐drug scaffold are reproducible across different tumor types that metastasize to bone.

It is important to note that eliminating well‐established bone metastases with immunotherapy alone is challenging, as bone metastases are typically “cold tumor” and often cannot be entirely resected due to anatomical constraints. Our approach leverages surgical debulking followed by an immunostimulatory scaffold to achieve local tumor control. In the models tested, the scaffold^MSA‐2^_αB7‐H3‐MP_ consistently induced robust immune activation and tumor regression in the postoperative setting.

These findings highlight the promise of this sequential dual‐immunotherapy scaffold as a strategy to improve outcomes for patients with bone metastatic disease in future clinical applications.

## 3 Discussion

Bone metastases predominantly occur in the axial skeleton, particularly the spine [3], and are frequently accompanied by complications such as pathological fractures and spinal cord compression, necessitating surgical intervention. However, due to anatomical proximity to critical structures like the spinal cord and major blood vessels, radical excision of bone lesions is generally unfeasible. This limitation contributes to a high incidence of local recurrence and further metastatic spread following surgery. Therefore, adjuvant therapeutic strategies are essential to eradicate residual tumor cells and improve long‐term outcomes.

In recent years, ICB has emerged as a transformative strategy in oncology, yet its efficacy against bone metastases remains limited. Bone metastases are prototypical “cold” tumors that fail to respond to ICB therapies, largely due to the abundance of MDSCs and TAMs in the bone marrow microenvironment. For effective antitumor responses, sufficient cytolytic CD8^+^ T cells are required in the TME, and this is dependent on the activation of type I IFN (IFN‐I) signaling in this context. The STING protein serves as a critical regulator of IFN‐I signaling and has been demonstrated to be essential for the efficacy of ICB [11]. Therefore, STING agonists have garnered widespread attention for their potential in antitumor immunotherapy. Recent advances in the development of non‐nucleotide STING agonists have addressed limitations associated with classical cyclic dinucleotide (CDN)‐based agonists, such as poor membrane permeability and enzymatic degradation [12, 44]. For example, MSA‐2, a potent small‐molecule STING agonist with oral bioavailability, demonstrates favorable characteristics, including enhanced cellular uptake in acidic TME and low systemic toxicity [13], with efficacy observed in multiple tumor models [15]. Nevertheless, a common challenge associated with STING agonists is the achievement of safe and effective drug delivery. The primary issue is that STING is a ubiquitously expressed protein across organs and cell types [11], which means systemic administration could lead to global host activation, potentially triggering a cytokine storm and organ toxicity [21]. Localized drug delivery represents an ideal strategy to avoid systemic toxicity [26, 27]. Notably, to maintain cellular homeostasis, the STING activation is negatively regulated by mechanisms involving STING protein degradation and autophagy [10]. As a result, single‐dose administration of STING agonists often fails to sustain durable immune responses. Additionally, studies have demonstrated that low doses, rather than high doses, of STING agonists prevent lymphocytes depletion in TME [45].

Thereby, we designed a freeze‐dried GelMA scaffold loaded with MSA‐2 for implantation into the bone metastasis site following resection. The scaffold enables controlled, sustained release of MSA‐2 to activate the STING‐IFNβ signaling in TME, and has shown a favorable safety profile. Furthermore, the GelMA scaffold possesses excellent tissue adhesion properties, and its porous structure facilitates lymphocytes chemotaxis and retention. Our experiment results confirmed an increase in cytotoxic CD8^+^ T cells following implantation of the scaffold. Interestingly, CyTOF analysis revealed elevated levels of activated NK cells. We focused our analysis on MDSCs and macrophages, the predominant immunosuppressive cell types in the bone metastatic TME. We found that the proportion of immunosuppressive MDSCs and M2 macrophages was reduced. Whereas polarizations of MDSCs into immunostimulatory cells and M1 polarization of intratumor macrophages (which have antigen‐presenting functions) exhibited opposite trends to increase. In the rechallenge model, we observed an increased proportion of mature DCs and memory T cells in the spleen, indicating systemic immune activation.

Interestingly, the role of the STING pathway in MDSCs remains controversial. Ping et al. and Eslam et al. found that STING activation in MDSCs enhances their immunosuppressive functions [46, 47]. In contrast, studies by Casey et al. and Chuan et al. suggested that STING activation contributes to myeloid repolarization and T cell activation, which were consistent with our observations [31, 48]. We speculate that these discrepancies may arise from differences in the methods and intensities used to activate the STING pathway across studies, warranting further investigation to elucidate the underlying mechanisms.

B7‐H3 (CD276), a member of the B7 immunoglobulin superfamily, is increasingly recognized as a key immunosuppressive checkpoint in the TME. Although initial studies reported B7‐H3's co‐stimulatory effects on cytotoxic T cell responses [49], accumulating evidence over the past decades has demonstrated its predominantly immunosuppressive role in the TME. B7‐H3 blockade enhances CD8^+^ T cell infiltration and functionality, ultimately leading to tumor suppression [34]. B7‐H3 overexpression has been observed in many types of tumors with relatively low PD‐L1 levels. For instance, the mRNA expression level of B7‐H3 is in the top 20% of genes expressed in prostate cancer, while PD‐L1 remains minimal [50]. Aberrant B7‐H3 overexpression in common malignancies (including breast, prostate, renal, and lung cancers that are prone to bone metastasis) correlates with poorer clinical outcomes and more aggressive disease progression in these patients [19, 20]. Crucially, B7‐H3 is highly expressed in tumors while remaining minimally expressed in normal tissues, making it an attractive and specific therapeutic target [51]. In addition, B7‐H3 has been extensively documented to promote tumor cell migration, proliferation, invasion, and drug resistance [20, 51]. Meanwhile, B7‐H3 could promote the M2 macrophage polarization [52], inhibit NK cell‐mediated antitumor functions [36], and induce the formation of neutrophil extracellular traps (NETs) [53]. Intriguingly, B7‐H3 blockade also inhibits osteoclast differentiation [54]. In conclusion, these findings suggest that B7‐H3 is an attractive target for bone metastases immunotherapy.

Beyond preventing T cell exhaustion in the context of prolonged STING activation, monoclonal antibodies targeting B7‐H3 exert tumor‐killing effects through antibody‐dependent cellular cytotoxicity (ADCC) mediated by NK cells and M1 macrophages [55, 56], both of which were notably enriched in the TME following our dual‐drug scaffold implantation. However, the receptor of B7‐H3 remains unclear, with TLT‐2, IL20RA, and PLA2R1 all being proposed candidates [20], a knowledge gap that currently limits broader therapeutic applications and warrants further investigation.

Combination therapies involving STING agonists and ICBs (PD‐1 antibodies) have demonstrated potent synergistic effects in multiple models. In these studies, STING agonists and ICBs are administered simultaneously [57]. Whereas, we designed a sequential delivery system for the STING agonist (MSA‐2) and ICB (αB7‐H3). This design is based on the following rationale: first, activation of the STING–IFNβ signaling pathway in TME is a prerequisite for ICB efficacy; second, STING pathway activation upregulates immune checkpoint molecules such as B7‐H3/PD‐L1 in TME, leading to accompanied immunosuppressive effects. Notably, our data identify B7‐H3 as an adaptive checkpoint induced by STING activation across both tumor and myeloid compartments, emerging with a defined temporal pattern and potentially restraining STING‐primed CD8^+^ T‐cell responses. This mechanistic insight provides a rationale for temporally staging B7‐H3 blockade after STING priming in the bone‐metastatic niche, rather than administering the two agents simultaneously. The sequential delivery of ICB could block upregulated immune checkpoint molecules, preventing T cell exhaustion. Indeed, our experimental results confirmed that prior delivery of the STING agonist followed by the αB7‐H3 elicited superior synergistic antitumor effects compared to simultaneous administration. The sequence dependence was supported by in vitro release profiling, which showed rapid MSA‐2 release but delayed, pH‐accelerated αB7‐H3 release from CaCO_3_ microparticles, together with in vivo imaging indicating prolonged local depot retention at the resection cavity. Importantly, in a reverse‐sequence control in which the release order was inverted, immune remodeling and therapeutic efficacy were attenuated, underscoring that the temporal sequencing of STING priming relative to B7‐H3 blockade is a key determinant of treatment outcome. This observation echoes the findings of Jeong et al., who reported that sequential treatment with STING agonist ADU‐S100 and immune checkpoint blockade (αOX40 and αPD‐1) alters downstream immune responses compared to co‐stimulation [58], underscoring the importance of timing and sequence in immunotherapy. Notably, tumor debulking was performed at an early stage in our models, whereas clinical bone metastases that require surgery typically represent more advanced lesions. Therefore, the immune microenvironment at this timepoint may not fully recapitulate that in surgically treated, late‐stage bone metastases. Future studies using more clinically representative models and/or higher‐resolution image guidance will be valuable in enabling more precise tumor resection and facilitating implantation of local therapeutic materials in a manner that more closely mirrors clinical practice. In addition, the effectiveness and safety of the scaffold^MSA‐2^_αB7‐H3‐MP_ should be further evaluated in large mammalian models before clinical application. Additionally, while the current loading doses and release kinetics of MSA‐2 and αB7‐H3 were supported by the release profiling and functional in vivo comparisons, further optimization may further improve the therapeutic window and translational robustness.

## 4 Conclusion

In summary, we developed an implantable GelMA‐based composite scaffold co‐loaded with αB7‐H3‐MPs and a non‐nucleotide STING agonist MSA‐2. This local delivery system is designed to reprogram the immunosuppressive bone tumor microenvironment and potentiate antitumor immunity following surgical tumor debulking. Upon implantation, the scaffold first releases MSA‐2, which activates STING signaling in tumor cells and immunosuppressive myeloid cells (MDSCs and macrophages), leading to a surge in IFNβ production and the recruitment/activation of cytotoxic CD8^+^ T cells. Subsequently, as the CaCO_3_ microparticles dissolve in the acidic tumor milieu, αB7‐H3 antibody is released, blocking the upregulated B7‐H3 checkpoint and preventing T cell exhaustion. Through comprehensive in vitro and in vivo experiments, we demonstrated that this sequential release approach significantly diminishes immunosuppressive cell populations (MDSCs and M2 macrophages) while boosting effector immune cells (M1 macrophages, effector T cells, NK cells), culminating in enhanced tumor cell killing. Mass cytometry and flow cytometry analyses confirmed that the scaffold induces a phenotypic shift in the tumor microenvironment from immune‐suppressive to immune‐activated, and amplifies the presence and activity of tumor‐attacking lymphocytes. Moreover, our scaffold elicited durable protective immunity, as evidenced by tumor rechallenge studies, and showed broad efficacy in multiple bone metastasis models. By integrating STING agonist therapy with immune checkpoint blockade in a spatiotemporally controlled manner, this dual‐drug scaffold maximizes therapeutic synergy while minimizing systemic toxicity. It represents a promising and readily translatable immunotherapeutic strategy for the management of bone metastases.

## 5 Experimental Section

### 5.1 Materials

All chemicals were used as received from commercial suppliers. Anhydrous calcium chloride (CaCl_2_) and sodium hydrogen carbonate (NaHCO_3_) were purchased from Macklin Biochemical Technology Co., Ltd. (Shanghai, China). Polyvinyl alcohol (PVA) copolymer was obtained from Sigma–Aldrich (Shanghai, China). Gelatin methacryloyl (GelMA) and the photoinitiator lithium phenyl‐2,4,6‐trimethylbenzoylphosphinate (LAP) were purchased from EFL‐Tech (Suzhou, China). Fluorescent tracers, including FITC‐labeled bovine serum albumin (BSA‐FITC) and 1,1'‐dioctadecyl‐3,3,3',3'‐tetramethylindocarbocyanine perchlorate (DiL), were obtained from Ruixi Biotech (Xi'an, China) and Beyotime Biotechnology (Shanghai, China), respectively. MSA‐2 was acquired from Topule (Wuhan, China). RPMI‐1640 medium, DMEM, and Trizol reagent (for total RNA isolation) were purchased from Thermo Fisher Scientific (USA). The anti‐B7‐H3 antibody (InVivoPlus anti‐mouse CD276, Catalog #BE0124) was obtained from BioXcell (USA).

### 5.2 Preparation and Characterization of αB7‐H3‐MPs

αB7‐H3‐MPs were prepared via a controlled precipitation method. Briefly, 1 mL of 100 mm CaCl_2_ in deionized water was slowly added under gentle agitation to 1 mL of deionized water containing 500 µg of B7‐H3 antibody, 10 mg of PVA, and 100 mm NaHCO_3_. The mixture was stirred at room temperature for 4 h to allow nucleation and growth of calcium carbonate crystals encapsulating the αB7‐H3 antibody. The resulting suspension was centrifuged (12 000 rpm, 5 min) and washed with PBS three times to remove unreacted ions, excess polymer, and unencapsulated antibody. The purified microparticles were then characterized for size, charge, morphology, and antibody encapsulation. Hydrodynamic diameter and zeta potential were measured using a particle size analyzer (90Plus/BI‐MAS, Brookhaven, USA). Microparticle morphology was observed via transmission electron microscopy (TEM) on a JEM‐1400 microscope (Hitachi, Japan). The antibody encapsulation efficiency within the CaCO_3_ microparticles was quantified by enzyme‐linked immunosorbent assay (ELISA).

### 5.3 Preparation and Characterization of αB7‐H3‐MPs & MSA‐2 Hydrogel Composite

To create a composite therapeutic scaffold, 20 mg of αB7‐H3‐MPs and 4 mg of the STING agonist MSA‐2 were dispersed in 1 mL of 10% (w/v) GelMA prepolymer solution containing 0.25% (w/v) LAP photoinitiator. The suspension was thoroughly mixed by vortexing and then aliquoted into a 96‐well plate mold. Photo‐crosslinking was carried out by exposing the mixture to 405 nm light (25 mW/cm^2^) for 15 s, yielding a solid hydrogel. The hydrogel was subsequently freeze‐dried to produce a porous scaffold suitable for implantation. The dried composite (scaffold^MSA‐2^_αB7‐H3‐MP_) was characterized for morphology, composition, and mechanical strength. Surface microstructure was examined by a field‐emission scanning electron microscope (FE‐SEM, Hitachi, SU8200) operating at an acceleration voltage of 3 kV. Elemental composition of the scaffold was analyzed using a scanning transmission electron microscope coupled with an energy‐dispersive X‐ray spectroscope (STEM‐EDS). Mechanical properties were evaluated using a universal testing machine (Instron 5943, USA), with compressive modulus determined through unconfined compression testing at a strain rate of 1 mm/min.

### 5.4 Cell Culture

To investigate biological effects in vitro, we employed several murine tumor cell lines and primary cell cultures. The 4T1 murine breast cancer cell line and the LLC murine Lewis lung carcinoma cell line were obtained from the Cell Collection of the Chinese Academy of Science (Shanghai, China). A Myc‐Cap murine prostate cancer cell line was purchased from MEISEN CTCC (CTCC‐001‐0359, China). All cell lines were cultured in DMEM supplemented with 10% (v/v) fetal bovine serum (FBS; Life Technologies) and 1% (w/v) penicillin‐streptomycin at 37°C in a humidified 5% CO_2_ incubator.

For treatment experiments, cells were exposed either to free MSA‐2 plus αB7‐H3 or to scaffold‐derived conditioned medium. To prepare the conditioned medium, the drug‐loaded GelMA scaffolds were placed in the upper chamber of Transwell inserts, with the lower chamber containing culture medium adjusted to pH 6.5 (to mimic the slightly acidic tumor microenvironment). The inserts were incubated for 48 h at 37°C, allowing released MSA‐2 and αB7‐H3 to diffuse into the lower compartment. Thereafter, the medium from the lower chamber was collected and centrifuged at 1000 × g for 10 min to remove any particulates. The clarified supernatant, containing the released drugs, was directly used to treat cells in subsequent assays.

### 5.5 Differentiation of BM‐MDSCs, BMDMs and BMDCs In Vitro

Bone marrow‐derived myeloid cells were generated to model immunosuppressive cells in the tumor microenvironment. Bone marrow was harvested from the tibiae of 6–8‐week‐old FVB mice. Marrow cells were flushed out with RPMI‐1640 medium containing 2% heat‐inactivated FBS, filtered through a 70‐µm cell strainer, and treated with red blood cell lysis buffer (BioLegend) for 2 min at room temperature to remove erythrocytes. After washing and centrifugation, the remaining leukocytes were resuspended in complete RPMI‐1640 medium.

For myeloid‐derived suppressor cell (MDSC) induction, bone marrow cells were cultured in RPMI‐1640 supplemented with 10% FBS, 40 ng/mL recombinant murine GM‐CSF (Peprotech, #315‐03), and 40 ng/mL recombinant IL‐6 (Peprotech, #216‐16). Cells were maintained at 37°C with 5% CO_2_ for 4 days, after which the resulting MDSCs were harvested for analysis.

For differentiation of bone marrow‐derived macrophages (BMDMs), bone marrow cells were cultured in complete RPMI‐1640 containing 10% FBS and 20 ng/mL recombinant murine M‐CSF (Peprotech, #315‐02). The medium was replenished every other day to ensure sufficient cytokine support. On day 5, adherent macrophages were stimulated with IL‐4 (20 ng/mL; PeproTech, #214‐14) for 24 h to polarize them toward the M2 phenotype. The polarized BMDMs were harvested on day 6 for downstream assays.

Bone marrow cells were cultured in RPMI‐1640 supplemented with 10% FBS, 40 ng/mL recombinant murine GM‐CSF (Peprotech, #315‐03), and 40 ng/mL recombinant murine IL‐4 (Peprotech, #214‐14) for 6 days at 37°C with 5% CO_2_. Fresh cytokine‐containing medium was replenished every 2–3 days. On day 6, loosely adherent and non‐adherent cells were collected as immature BMDCs. For maturation assays, BMDCs were incubated for 48 h with conditioned medium prepared as described above. BMDC maturation was assessed by flow cytometry based on CD80, CD86, and MHC class II expression within the CD11c^+^ population.

### 5.6 RNA Extraction, Reverse Transcription, and Real‐Time Quantitative PCR (qRT‐PCR)

Gene expression analysis was performed as previously described [40]. Total RNA was extracted from cells using TRIzol reagent (Invitrogen). The isolated RNA (2 µg per sample) was reverse‐transcribed into cDNA using the Color Reverse Transcription Kit (EZBioscience) according to the manufacturer's instructions. The resulting cDNA was then used as a template for quantitative PCR with a 2 × Color SYBR Green qPCR Master Mix (EZBioscience) on a CFX96 system (Bio‐Rad, USA). β‐Actin served as the internal reference gene for normalization. Primer sequences for target genes are provided in Table S1. Relative mRNA expression levels were calculated using the comparative 2‐ΔΔCt method.

### 5.7 Western Blotting

Protein‐level changes in signaling pathways were examined by Western blotting. MDSCs, BMDMs, or Myc‐Cap tumor cells (3 × 10^6^ per well) were seeded in 6‐well plates and cultured for 24 h. Cells were then treated with the conditioned medium for 48 h (as described in the section of Cell Culture). After treatment, cells were lysed in RIPA buffer, and the total protein concentration was determined using a BCA assay. Equal amounts of protein were separated by SDS‐PAGE and transferred to PVDF membranes. Western blotting was performed following standard protocols, as previously described [40]. The membranes were probed with primary antibodies against STING (Cell Signaling Technology, #50494, 1:1000), phosphorylated STING (Cell Signaling Technology, #72971, 1:1000), IRF3 (Cell Signaling Technology, #4302, 1:1000), phosphorylated IRF3 (Cell Signaling Technology, #29047, 1:1000), and B7‐H3 (Abclonal, A17216, 1:1000). β‐Actin (Cell Signaling Technology, #4967, 1:1000) was used as an internal control. After incubation with HRP‐conjugated secondary antibodies, protein bands were detected using Bio‐Rad ChemiDoc Imaging System.

### 5.8 T Cell Proliferation Assay

To assess T Cell proliferation, CD8^+^ T lymphocytes were isolated from the spleens of FVB mice using a Mouse CD8^+^ T Cell Isolation Kit (Stemcell Technologies, #19853, Vancouver, Canada). Purified T cells were labeled with carboxyfluorescein succinimidyl ester (CFSE, 2 µM, Abmole). To activate the T cells, 24‐well plates were pre‐coated with anti‐CD3 (1 µg/mL, BioLegend) and anti‐CD28 (1 µg/mL, BioLegend) antibodies overnight at 4°C (uncoated wells served as controls). CFSE‐labeled CD8^+^ T cells were first cultured in these coated wells for 48 h to induce activation.

For the proliferation assay, treated MDSCs (prepared as described above and exposed to conditioned medium for 48 h) were co‐cultured with the pre‐activated CFSE‐labeled CD8^+^ T cells at a 1:1 ratio. After 48 h of co‐culture, T cell proliferation was evaluated by flow cytometry as indicated by CFSE dilution (CytoFLEX Flow Cytometer, Beckman, USA), following established protocols [59].

Additionally, to analyze the combined effect of myeloid and tumor cells on T cell activity, a co‐culture system was set up with Myc‐Cap tumor cells, MDSCs, and BMDMs. These three cell types were pre‐treated with the conditioned medium for 48 h and then co‐cultured together at a 1:1:1 ratio for another 48 h. Subsequently, CFSE‐labeled, pre‐activated CD8^+^ T cells (prepared as above) were added to the mixed culture at a ratio of 1:1:1:1 (tumor:MDSC:BMDM:T cell). This four‐cell co‐culture was maintained for 48 h to allow immune cell–tumor cell interactions.

### 5.9 Animal Experiments

In vivo experiments were conducted to evaluate the therapeutic efficacy and immune effects of the treatment in a bone metastasis model. Male FVB/NJ and C57BL/6J mice, as well as female BALB/c mice (GemPharmatech, Jiangsu, China), were used for the different tumor models. All mice were 6–8 weeks old and were housed in a specific pathogen‐free (SPF) facility (≤ 5 mice per cage) at approximately 25°C and 50% humidity, under a 12 h light/dark cycle with food and water provided ad libitum. All animal procedures were approved by the Institutional Animal Care and Use Committee (IACUC) of Sun Yat‐sen University (approval NO. SYSU‐IACUC‐2025‐000394), and experiments were performed in accordance with institutional and national guidelines.

A bone metastasis tumor model was established by intratibial injection of tumor cells. Mice were anesthetized with isoflurane, and the hindlimb was sterilized after fur removal. A 29‐gauge insulin syringe (BD) was inserted through the tibial plateau (via the patellar ligament) into the medullary cavity. Tumor cells (10 µL of cell suspension) were slowly injected into the tibia. For Myc‐Cap (prostate cancer) and LLC (lung cancer) models, 1 × 10^6^ cells were injected per tibia. For the 4T1 (breast cancer) model, 2 × 10^5^ cells were injected. After injection, the needle was withdrawn, and gentle pressure was applied to the injection site to prevent reflux and bleeding. Mice were monitored during recovery and were checked daily for signs of limb pain or distress.

After allowing tumor establishment, a surgical resection of the primary intratibial tumor was performed to mimic a clinical scenario of incomplete tumor removal. Under anesthesia and sterile conditions, an incision was made to expose the tibia, and the bulk of the tumor within the bone was surgically curetted. All procedures during curettage and subsequent saline irrigation were performed gently and carefully to avoid inadvertent breach of the contralateral tibial cortex and to avoid major vessels, thereby minimizing perioperative complications (e.g., embolic events including pulmonary embolism, fracture, and excessive bleeding). This debulking procedure left a small residual tumor to simulate post‐surgical minimal residual disease. Successful partial resection was confirmed by in vivo bioluminescence imaging (BLI): the luminescent signal from the tibia was reduced by at least 90% compared to pre‐surgery levels, indicating that most tumor cells had been removed. Only mice meeting this criterion were advanced to subsequent treatment and analysis. Immediately after confirmation, mice were randomly assigned to control or treatment groups (6 mice per group). After intratibial tumor curettage, the tibial medullary cavity was gently irrigated with sterile saline to remove loose tissue fragments and blood before local application of the free MSA‐2 + αB7‐H3 solution or the scaffold. Treatments were applied locally at the surgery site before wound closure. For the drug combination control, a solution of free MSA‐2 plus αB7‐H3 was prepared by dissolving MSA‐2 in 1% (w/v) carboxymethylcellulose (CMC) solution via ultrasonication and then mixing in αB7‐H3 antibody once the solution cooled to room temperature. This free drug solution (MSA‐2 + αB7‐H3) was injected into the tibial cavity. For the scaffold treatment, a pre‐fabricated scaffold^MSA‐2^_αB7‐H3‐MP_ was implanted into the resection cavity. The muscle and skin were then sutured, and the wound area was disinfected.

Tumor recurrence and progression were monitored over time using an IVIS Spectrum in vivo imaging system (PerkinElmer). Mice were injected intraperitoneally with D‐luciferin (100 µL of 15 mg/mL solution, Invitrogen, #L2916) 10 min before imaging. Under isoflurane anesthesia, bioluminescent images of the mice were acquired at weekly intervals post‐surgery. Tumor burden in the tibia was quantified as the average radiance (photons/sec/cm^2^/sr) within a fixed region of interest over the tibial area, using Living Image software (PerkinElmer). For each mouse, the radiance was normalized to the signal measured on day 0 (immediately after surgery) to track relative tumor growth. Mice were observed daily, and those that reached humane endpoints (e.g., >10% loss of baseline body weight, paralysis, or head tilting, or severe distress) were humanely euthanized.

To evaluate long‐term antitumor immunity, a tumor rechallenge experiment was performed. Mice that achieved complete tumor regression after treatment with the scaffold^MSA‐2^_αB7‐H3‐MP_ were kept for an additional 2 weeks. On day 21 post‐surgery, these tumor‐free mice (along with age‐matched naïve control mice) were rechallenged by injecting Myc‐Cap tumor cells (1 × 10^6^ cells in 10 µL PBS) into the contralateral tibia, following the same intratibial injection procedure as before. Tumor growth in the rechallenged tibiae was monitored by in vivo bioluminescence imaging (BLI) at defined time points. The development of bioluminescent signal in previously treated mice vs naïve mice was compared to evaluate the presence of immunological memory. Mice were closely observed and euthanized upon showing any humane endpoint criteria.

### 5.10 Statistical Analysis

All quantitative data are presented as mean ± standard deviation (SD) unless stated otherwise, with error bars representing the SD. Unless specified otherwise, the mean and SD were calculated from three or more independent replicates. Statistical analysis was performed using GraphPad Prism 8.0.2 (GraphPad Software, USA). For comparisons between two groups, an unpaired two‐tailed Student's *t*‐test was applied for data following a normal distribution, while the Mann‐Whitney *U* test was applied for non‐normally distributed data. Comparisons among three or more groups were assessed using one‐way analysis of variance (ANOVA), followed by Tukey's post–hoc test was conducted. For comparisons involving three or more groups with two independent categorical variables, two‐way ANOVA with Tukey's multiple comparisons test was applied. Survival curves were analyzed using the log‐rank (Mantel–Cox) test. Statistically significant differences were indicated as follows: n.s., not significant; ^*^ *p* < 0.05, ^**^ *p* < 0.01, and ^***^ *p* < 0.001 (Scheme 1).

## Conflicts of Interest

The authors declare no conflicts of interest.

6

## Supporting information




**Supporting File**: advs74521‐sup‐0001‐SuppMat.pdf.

## Data Availability

The data that support the findings of this study are available from the corresponding author upon reasonable request.
