# Supplementary material for: An Implantable Scaffold Sequentially Releasing STING Agonist and B7‐H3 Antibody for Bone Metastasis Immunotherapy
Source: Adv Sci (Weinh). 2026 Feb 25;13(33):e20642. doi: 10.1002/advs.202520642 (PMC13271628; doi:10.1002/advs.202520642)
Supplement: Supplementary file 1 — Supporting File: advs74521‐sup‐0001‐SuppMat.pdf. [file ADVS-13-e20642-s001.pdf]

Supporting Information

An Implantable Scaffold Sequentially Releasing STING Agonist and B7-H3 Antibody for Bone Metastasis Immunotherapy

Qijun Lin, Hong Xiao, Shuai Fan, Kaimin Cai, Yanteng Xu, Xinwen Wang, Guanhong Chen, Chuandong Lang, Xinsheng Peng*, Mingqiang Li*, and Yuhu Dai**

Q. Lin, S. Fan, G. Chen, X. Peng, Y. Dai
Department of Orthopedic Surgery
The First Affiliated Hospital
Sun Yat-sen University
Guangzhou 510080, China
E-mail: pengxsh@mail.sysu.edu.cn; daiyh5@mail.sysu.edu.cn

Q. Lin, S. Fan, G. Chen, X. Peng, Y. Dai
Guangdong Provincial Key Laboratory of Orthopedics and Traumatology
Guangzhou 510080, China

X. Hong, K. Cai
Department of Medical Ultrasound
Laboratory of Novel Optoacoustic (Ultrasonic) Imaging
The Third Affiliated Hospital
Sun Yat-sen University,
Guangzhou 510630, China

Y. Xu, M. Li
Laboratory of Biomaterials and Translational Medicine
Center for Nanomedicine
The Third Affiliated Hospital
Sun Yat-sen University
Guangzhou 510630, China
E-mail: limq567@mail.sysu.edu.cn

X. Wang, C. Lang
Department of Orthopedics
The First Affiliated Hospital of USTC
Division of Life Sciences and Medicine
University of Science and Technology of China
Hefei, Anhui 230001, China
E-mail: langchd@ustc.edu.cn

M. Li
Cell-Gene Therapy Center

Institute for Frontier Interdisciplinary Research in Health Sciences and Technology
Sun Yat-sen University
Guangzhou 510080, China

M. Li
Key Laboratory for Polymeric Composite and Functional Materials of Ministry of Education
Sun Yat-sen University
Guangzhou 510275, China

Experimental Section

In Vitro and In Vivo Drug Release: The release kinetics of αB7-H3 and MSA-2 from the hydrogel scaffolds were evaluated both in vitro and in vivo. For in vitro release, scaffold samples were incubated in PBS (pH 7.4 or pH 6.5, 37 °C) to simulate physiological and mildly acidic tumor microenvironment conditions. At predetermined time points over 72 h, aliquots were withdrawn and replaced with fresh PBS. The amount of αB7-H3 released was quantified using a rat IgG ELISA kit (PI482, Beyotime), while MSA-2 release was measured by high-performance liquid chromatography (HPLC).

For in vivo release assessment, fluorescently labeled surrogates were used to visualize payload distribution. Hydrogel scaffold containing BSA-FITC-loaded $CaCO_3$ microparticles and DiL were prepared and imaged under a Nikon Eclipse Ti fluorescence microscope equipped with a Nikon DS-Qi2 monochrome camera (version 5.21.00; Nikon, Tokyo, Japan) to confirm uniform loading. These fluorescently loaded scaffolds were then implanted into the intratibial tumor resection site in FVB mice. The spatial and temporal release of payload was monitored non-invasively by capturing DiL (red) and FITC (green) signals weekly using an IVIS Spectrum in vivo imaging system (Perkin Elmer).

Tumor Cell Apoptosis Assay: To assess immune-mediated tumor cell killing in vitro, we measured apoptosis of tumor cells after co-culture with activated T cells. $CD8^+$ T cells were isolated and activated as described above, with an extended activation period of 4 days on anti-CD3/anti-CD28-coated plates to ensure robust cytotoxic function. In parallel, MDSCs, BMDMs, and Myc-Cap-GFP tumor cells were co-cultured at a 1:1:1 ratio and pre-treated with the conditioned medium for 48 h. Subsequently, activated $CD8^+$ T cells were added to this pre-conditioned co-culture system at a ratio of 1:1:1:1 (tumor:MDSC:BMDM:T cell). The mixed culture was incubated for an additional 48 h to allow T cells to engage and induce tumor cell death.

Tumor cell apoptosis was then evaluated by flow cytometry using an Annexin V-APC/PI Apoptosis Detection Kit (Vazyme). After 48 h of co-culture, all cells in the co-culture were collected and washed twice with ice-cold PBS. Cells were resuspended in 100 μL of 1 × binding buffer, and 5 μL of Annexin V-APC and 5 μL of propidium iodide (PI) solution were added. Samples were gently mixed and incubated for 10 minutes at room temperature in the dark. Finally, 400 μL of binding buffer was added to each sample, and the cells were immediately analyzed on a CytoFLEX flow cytometer (Beckman Coulter, USA). Data were processed with FlowJo software (version 10.6.2, BD, USA).

Immunofluorescence Staining of p-IRF3: To visualize activation of the STING pathway at the cellular level, immunofluorescence staining for phosphorylated IRF3 (p-IRF3) was performed on treated tumor cells. Myc-Cap cells were grown on sterilized glass coverslips in 24-well plates until ~70% confluent and then treated under the desired. After treatment, cells were rinsed in PBS and fixed with 4% paraformaldehyde for 15 min. Fixed cells were permeabilized with 0.1% Triton X-100 in PBS for 20 min, followed by blocking with 5% bovine serum albumin (BSA) for 30 minutes at room temperature. Cells were then incubated overnight at 4°C with a primary antibody against p-IRF3 (Cell Signaling Technology, #29047, 1:250). The next day, samples were washed and incubated with an Alexa Fluor 488-conjugated secondary antibody (goat anti-mouse IgG (H+L), Cell Signaling Technology, #4408, 1:500) for 1 h at room temperature. Nuclei were counterstained with DAPI (Sigma-Aldrich) for 30 min. Finally, coverslips were mounted onto glass slides using an antifade mounting medium. Fluorescence images were captured with a Nikon Eclipse Ti inverted fluorescence microscope equipped with a DS-Qi2 monochrome camera (Nikon, Japan).

Mass Cytometry Analysis of the tumor microenvironment: High-dimensional mass cytometry (CyTOF) was used to profile immune cells in the tumor microenvironment following treatment. At the endpoint, bone marrow cells were collected from tumor-bearing tibias of mice. The tibias were flushed with cold Hanks' Balanced Salt Solution (HBSS) using a syringe with a 26G needle to obtain single-cell suspensions. Red blood cells were removed by treatment with lysing buffer (BD Biosciences, #555899), and the remaining cells were washed and counted. For CyTOF staining, cells were first incubated with Cell-ID Cisplatin-194Pt for 5 min and then blocked with blocking solution for 20 min on ice. After incubation, immune cells were stained for 30 min at room temperature with a panel of metal-conjugated antibodies targeting 42 surface or intracellular markers (antibody panel detailed in Table S1, Supporting Information). After staining, cells were washed, fixed, and analyzed on a Helios mass cytometer (Fluidigm, USA). The CyTOF data were normalized and then analyzed using FlowJo (version 10). Single, intact live cells were selected, and $CD45^+$ immune cells were identified. The x-shift algorithm was employed for cell subgroup clustering, annotation, t-SNE dimensionality reduction visualization, and statistical analysis. Cell populations were annotated based on marker expression, and frequencies of each cluster were compared between treatment groups.

Flow cytometric analysis: In parallel with CyTOF profiling, conventional flow cytometry was performed to quantify specific immune cell subsets in the bone marrow and spleen. Tumor-bearing tibiae and spleens were collected from mice at defined time points or endpoints. Single-cell suspensions from spleens were prepared by mechanical dissociation (mashing through a

70-μm strainer) in RPMI-1640 containing 2% FBS, while bone marrow cells were obtained by flushing the tibiae with RPMI-1640 containing 2% FBS. Red blood cells were lysed using an ammonium chloride-based lysis buffer before antibody staining. After centrifugation and resuspension, single cells were added to 5 mL flow cytometry tubes and blocked with anti-CD16/CD32 antibodies (Biolegend, #101302) before being stained for flow cytometry. To sort living cells, 7-AAD (7-aminoactinomycin D) viability dye (BioLegend, #420403) was added according to the manufacturer's protocol. Dead cells exhibiting compromised membrane integrity were identified as 7-AAD-positive and excluded from subsequent gating strategies.

The following antibody panels were used to identify immune cell populations (all antibodies from BioLegend unless otherwise noted). For analysis of MDSCs, FITC-conjugated anti-CD45 (#157214), PE/Cyanine7-conjugated anti-CD11b (#101216), BV510-conjugated anti-Gr-1 (#108457), and PE-conjugated anti-Arginase-1 (#165803) were used. For analysis of macrophage, FITC-conjugated anti-CD45 (#157214), PE/Cyanine7-conjugated anti-CD11b (#101216), BV605-conjugated anti-F4/80 (#123133), BV510-conjugated anti-CD86 (#105039), and PE-conjugated anti-CD206 (#321106) were used. For analysis of $CD3^+$ T cells, FITC-conjugated anti-CD45 (#157214), PE/Cyanine7-conjugated anti-CD11b (#101216), APC/Cyanine7-conjugated anti-CD3 (#100222), BV510-conjugated anti-CD4 (#100553), BV650-conjugated anti-CD8 (#100741), BV421-conjugated anti-CD107α (#121617) were used. For analysis of mature DCs, FITC-conjugated anti-CD45 (#157214), PE/Cyanine7-conjugated anti-CD11b (#101216), BV785-conjugated anti-CD11c (#117335), BV510-conjugated anti-CD86 (#105039), BV605-conjugated anti-CD80 (#105039), PE-conjugated anti-MHCII (#107607) were used. For analysis of T_{CM} ($CD44^+CD62L^+$) and T_{EM} ($CD44^+CD62L^-$), FITC-conjugated anti-CD45 (#157214), PE/Cyanine7-conjugated anti-CD11b (#101216), APC/Cyanine7-conjugated anti-CD3 (#100222), BV650-conjugated anti-CD8 (#100741), APC-conjugated anti-CD44 (#103011), PE-conjugated anti-CD62L (#161203) were used. For the in vitro co-culture experiment, PE-conjugated anti-CD276 (#135605) was used to detect the surface expression of B7-H3 on three cell types: MDSCs, BMDMs, and Myc-Cap cells. Stained samples were analyzed on a CytoFLEX flow cytometer (Beckman), and data were processed with FlowJo software (version 10.0.7, Tree Star).

Micro-CT Analysis: Micro-computed tomography (micro-CT) was used to assess bone destruction and regeneration in the tumor-bearing limbs, following a published protocol[46]. Briefly, mice were euthanized at the experimental endpoint, and the hind limbs were harvested. Tibiae were fixed in 4% paraformaldehyde for at least 48 h, then scanned using a high-resolution micro-CT scanner (SkyScan 1276, Bruker, Belgium). The acquired images were

reconstructed into 3D datasets using NRecon software (Bruker). Multiplanar cross-sectional views (coronal, sagittal, and transverse) were examined using Dataviewer software. For quantitative analysis, cortical bone was digitally segmented out to isolate the trabecular bone compartment. A consistent volume of interest (~1 mm^3 region, starting from the metaphysis) was defined in the trabeculae region of the tibia. The bone-related measurements were then quantified and analyzed using CTAn and CTVol software.

Histologic Evaluation: Histological and immunohistochemical analyses were performed on bone tissue sections to evaluate tumor cell viability, apoptosis, and proliferation. Resected tibiae were fixed in 4% paraformaldehyde, then decalcified in 5% EDTA solution for one week. After decalcification, specimens were processed and embedded in paraffin. Sections (5–7 μm thick) were cut from the paraffin blocks for staining.

For general histopathology, sections were stained with hematoxylin and eosin (H&E) to assess overall tissue morphology and the presence of residual tumor cells. Apoptotic cells were detected using the terminal deoxynucleotidyl transferase dUTP nick-end labeling (TUNEL) assay (Servicebio) following the manufacturer's protocol. For proliferative activity, immunofluorescent staining for Ki67 was carried out. Sections underwent deparaffinization, rehydration, and antigen retrieval, then were incubated with Ki67 Rabbit pAb (Cell Signaling Technology, #12202, 1:400). Following this, the sections were stained using the PANO Reagents PPD520, according to the protocol outlined in the Multi-fluorescence Immunohistochemistry Kit 4 Color TSA-Rab-275 (Panovue, #10079100020). Nuclei were counterstained with DAPI (included in the kit). Fluorescence images of the stained tissue sections were captured using an inverted fluorescence microscope (Ti2-U, Nikon), and the images were subsequently quantified using the Fiji software.

Biosafety Evaluation: The systemic biocompatibility of the treatments was assessed via serum biochemistry and histopathology. Blood samples were collected from mice at the experimental endpoint and allowed to clot, and serum was separated by centrifugation (1000 g, 20 min, 4 °C). Key indicators of organ function (e.g., liver enzymes, renal function markers) were measured using an automated biochemical analyzer (Hitachi 3100, Japan). Additionally, major organs (heart, liver, spleen, lungs, and kidneys) were harvested and fixed in 4% paraformaldehyde. Tissues were embedded in paraffin, sectioned, and stained with H&E. The histology of these organs was examined under a light microscope for any signs of toxicity, inflammation, or abnormal pathology.

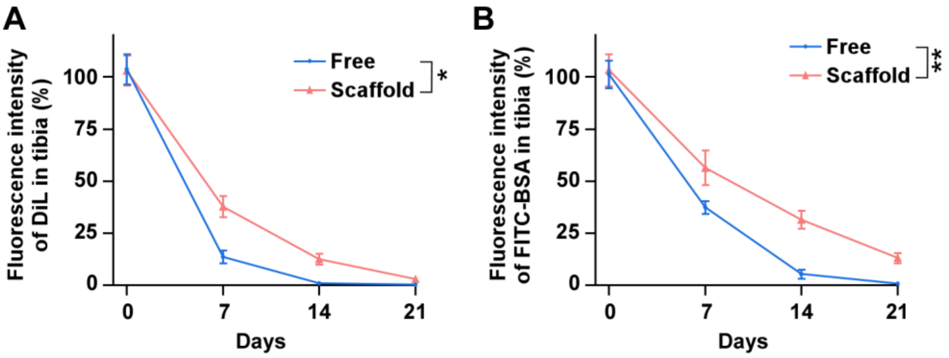

Figure S1. In vivo fluorescence intensity of (A) DiL and (B) FITC-BSA in the implanted scaffold over time. Free DiL and FITC-BSA served as controls. $*p < 0.05$, and $**p < 0.01$.

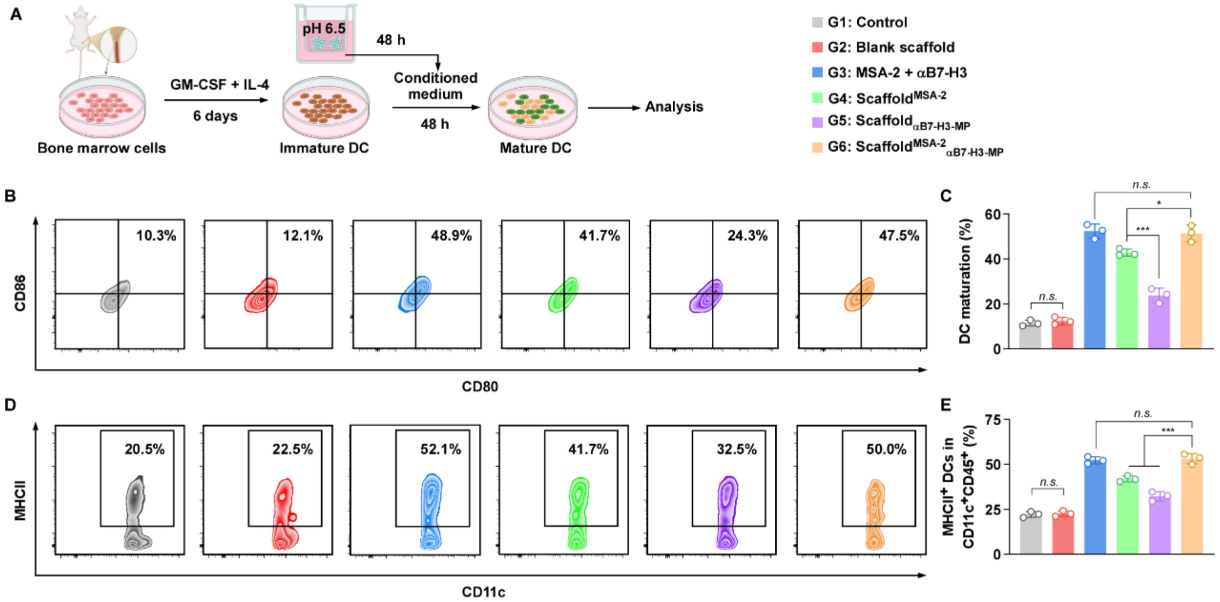

Figure S2. The drug-loaded GelMA scaffolds induce STING pathway activation and immune modulation in bone marrow–derived dendritic cells (BMDCs) in vitro. A) Schematic of BMDCs isolation, polarization, treatment, and analysis. B–E) Representative flow cytometry plots (B, D) and quantification (C, E) of matured DCs (B, C), and MHCII^{+} DCs (D, E) in BMDCs upon different treatments (n = 3 per group). n.s., not significant; *$p < 0.05$, and ***$p < 0.001$.

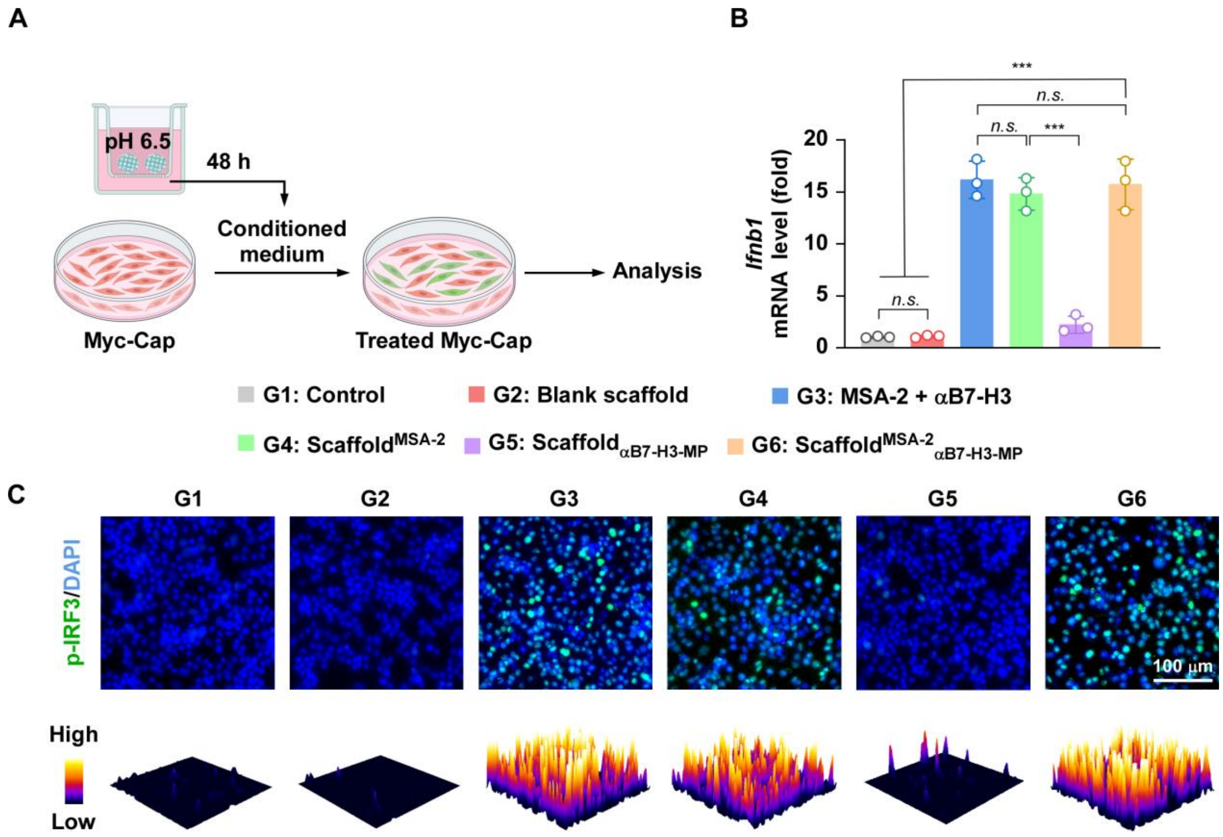

Figure S3. STING-IFNβ pathway activation in Myc-Cap tumor cells in vitro. (A) Schematic overview of the experimental design showing the treatments applied to Myc-Cap cells. (B) Relative *Ifnb1* mRNA expression in Myc-Cap cells after 6 h of treatment (n = 3 independent experiments). (C) Representative immunofluorescence images and corresponding 3D surface plots showing the fluorescence intensity of p-IRF3 in Myc-Cap cells after 6 h of treatment. n.s., not significant; ***, $p < 0.001$.

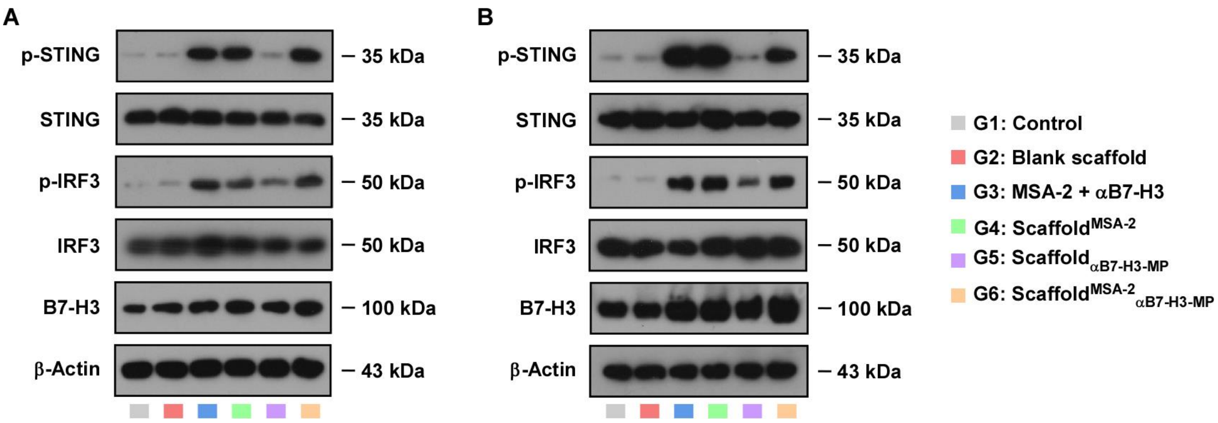

Figure S4. Representative western blot analysis of STING pathway proteins (STING, phospho-STING, IRF3, phospho-IRF3) and B7-H3 expression in BMDMs (A) and Myc-Cap cells (B).

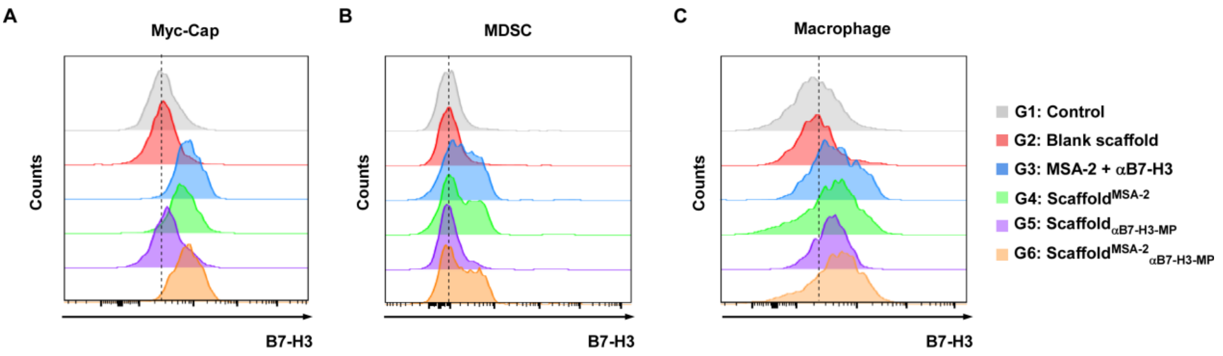

Figure S5. Representative flow cytometry histograms showing surface B7-H3 expression in Myc-Cap (A), MDSC (B), and BMDM (C) cells after 48 h of treatments (n = 3 independent experiments).

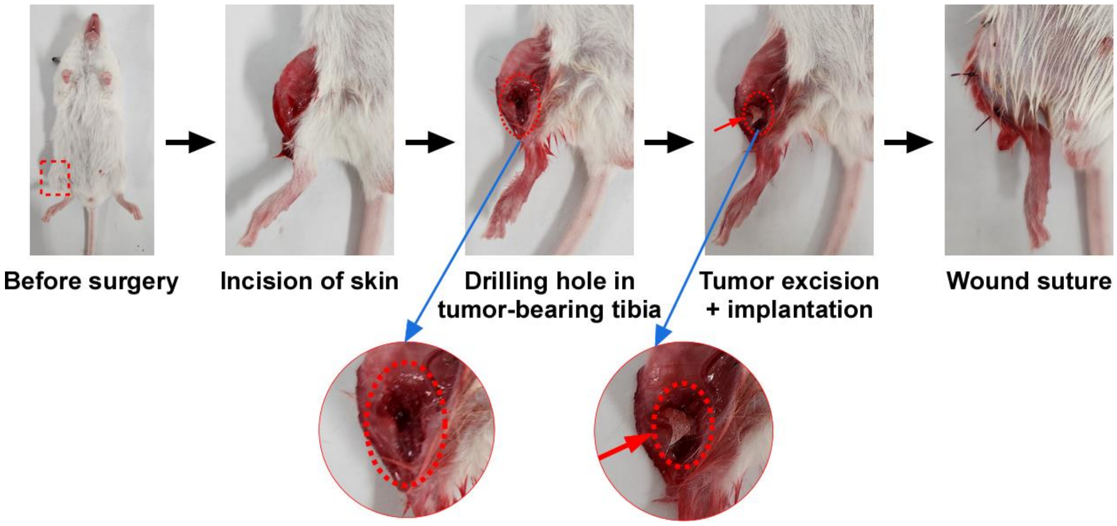

Figure S6. Illustration of tumor excision and subsequent implantation of GelMA scaffold in tumor-bearing FVB mice. Red arrows and circle highlight the location of the implanted GelMA scaffold.

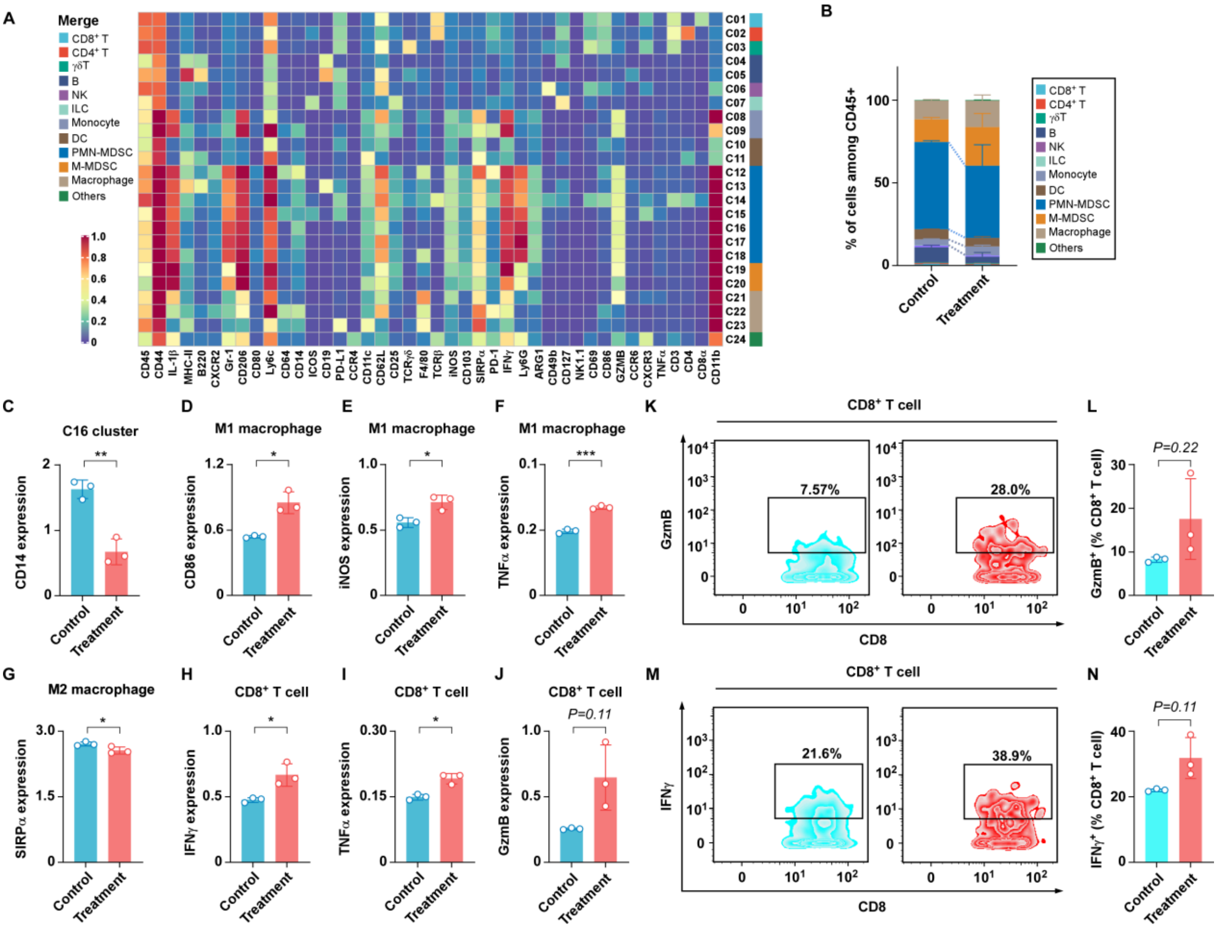

Figure S7. Scaffold$^{MSA\text{-}2}{}_{\alpha B7\text{-}H3\text{-}MP}$ remodels the tumor microenvironment. (A) The heatmap showing the expression levels of selected markers for 24 cell clusters. Red: high expression; Purple: low expression. (B) The proportional distribution of the distinct immune cell subtypes ($n = 3$ per group). (C–J) Statistical comparison of marker expression between two groups within specific immune cell subtypes (n = 3 per group). (K–N) CyTOF analysis with manual gating showing representative zebra plot (K, M) and quantification (L, N) of GzmB^{+} (K, L) and IFNγ^{+} CD8^{+} T cells (M, N) ($n = 3$ per group). *, $p < 0.05$; **, $p < 0.01$; ***, $p < 0.001$.

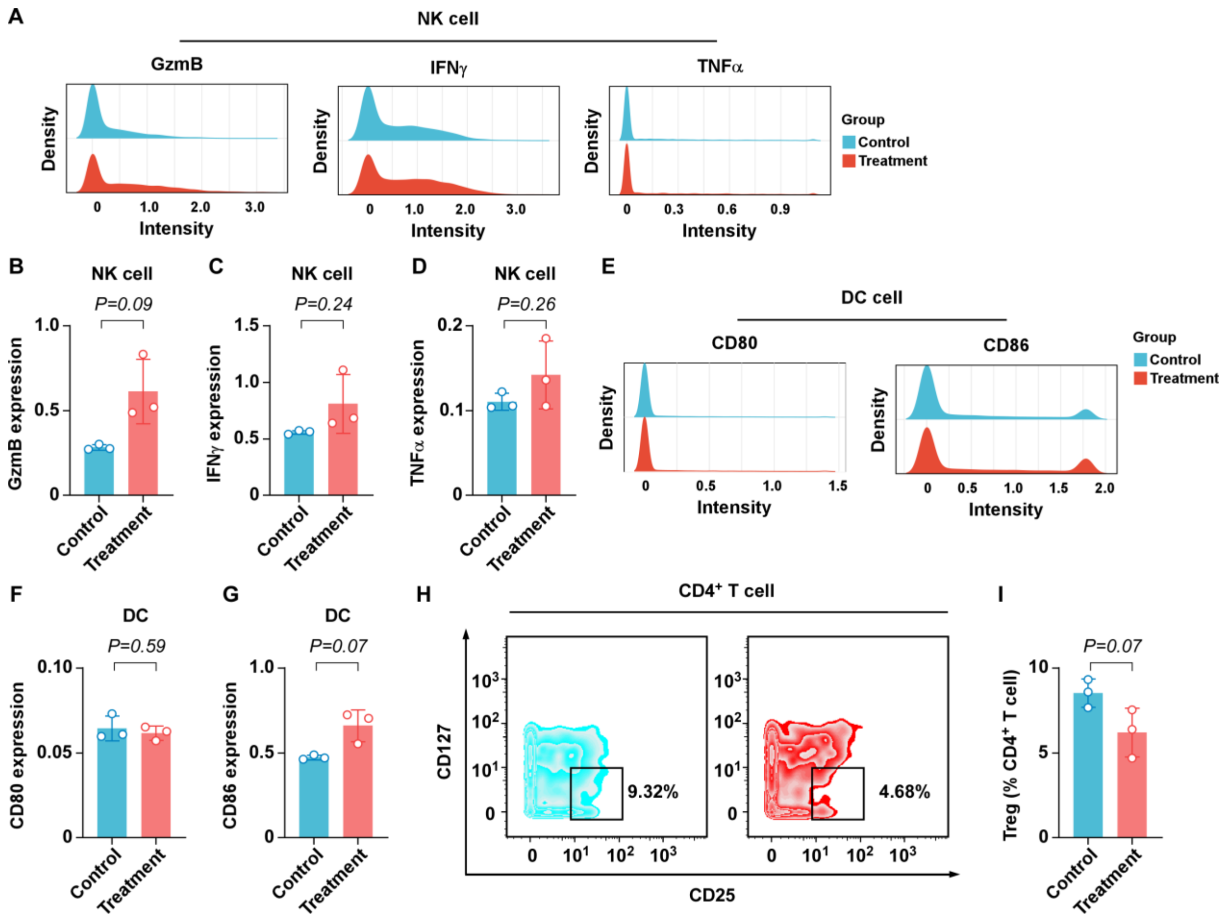

Figure S8. Early immunomodulatory effects of Scaffold$^{MSA\text{-}2}{}_{\alpha B7\text{-}H3\text{-}MP}$ on other immune cell populations. (A) Differential expression density of GzmB, IFNγ, and TNFα proteins in NK cells. (B–D) Statistical comparison of marker expression in NK cells (n = 3 per group). (E) Differential expression density of mature markers CD80 and CD86 in DCs. (F, G) Statistical analysis of CD80 and CD86 expression in DCs (n = 3 per group). (H, I) CyTOF data with manual gating showing representative zebra plots (H) and quantification (I) of Treg cells (n = 3 per group).

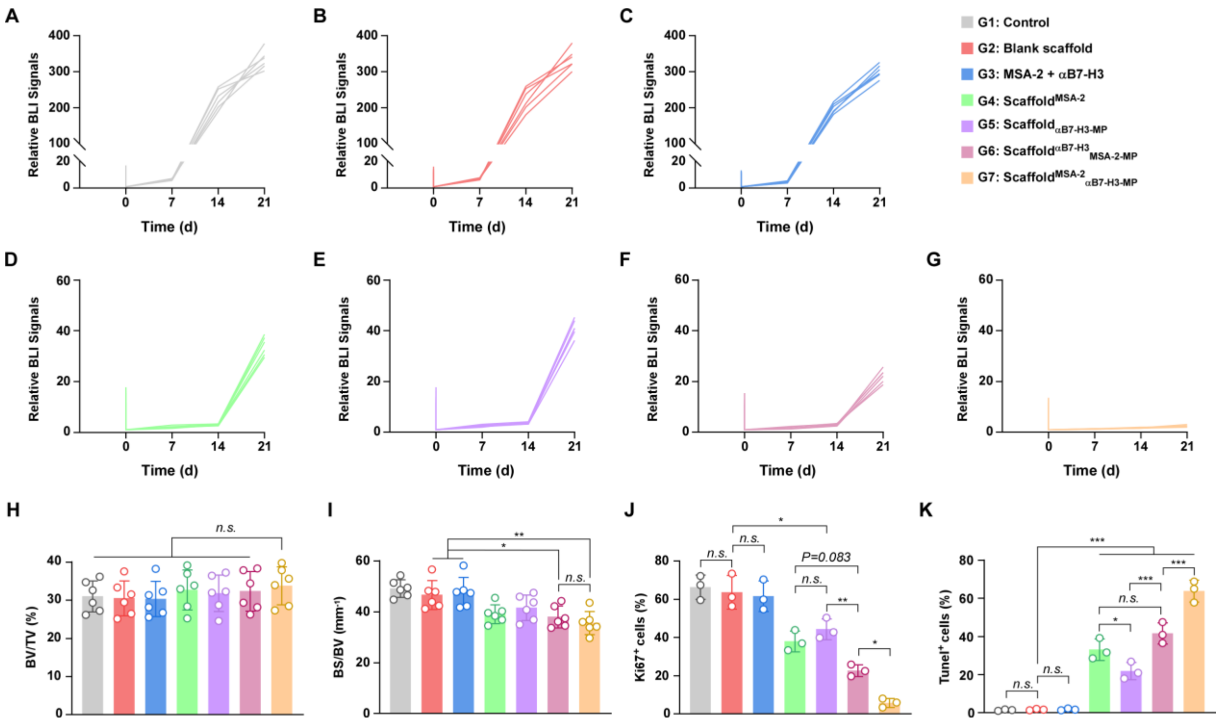

Figure S9. In vivo suppression of tumor recurrence. (A–G) Individual bioluminescence imaging (BLI) data showing relative signals from tibial lesions in each mouse across all treatment groups (n = 6 per group). (H, I) Quantitative analysis of bone parameters in intratibial lesions (n = 6 per group). BV/TV, bone/tissue volume ratio. BS/BV, bone surface/volume ratio. (J, K) Quantification of $Ki67^+$ and $TUNEL^+$ cells in tumor sections (n = 3 per group). n.s., not significant; *, $p < 0.05$; **, $p < 0.01$; ***, $p < 0.001$.

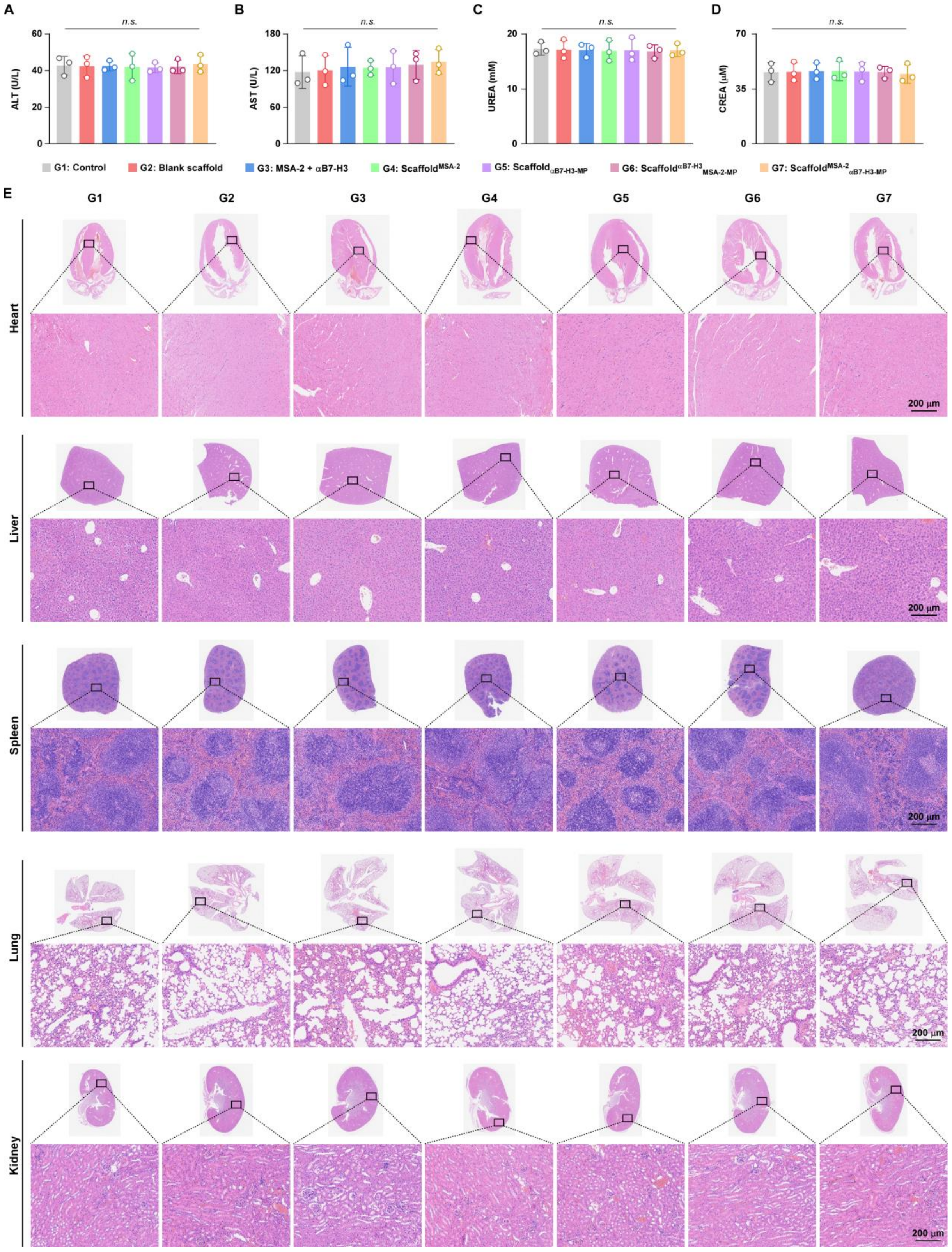

Figure S10. Biocompatibility assessment of GelMA scaffold in vivo. (A–D) Serum levels of ALT, AST, UREA, and CREA in treated mice, demonstrating normal liver (ALT, AST) and kidney (UREA and CREA) function (n = 3 mice). (E) Representative H&E staining of heart, liver, spleen, lung, and kidney tissues after treatment. n.s., not significant.

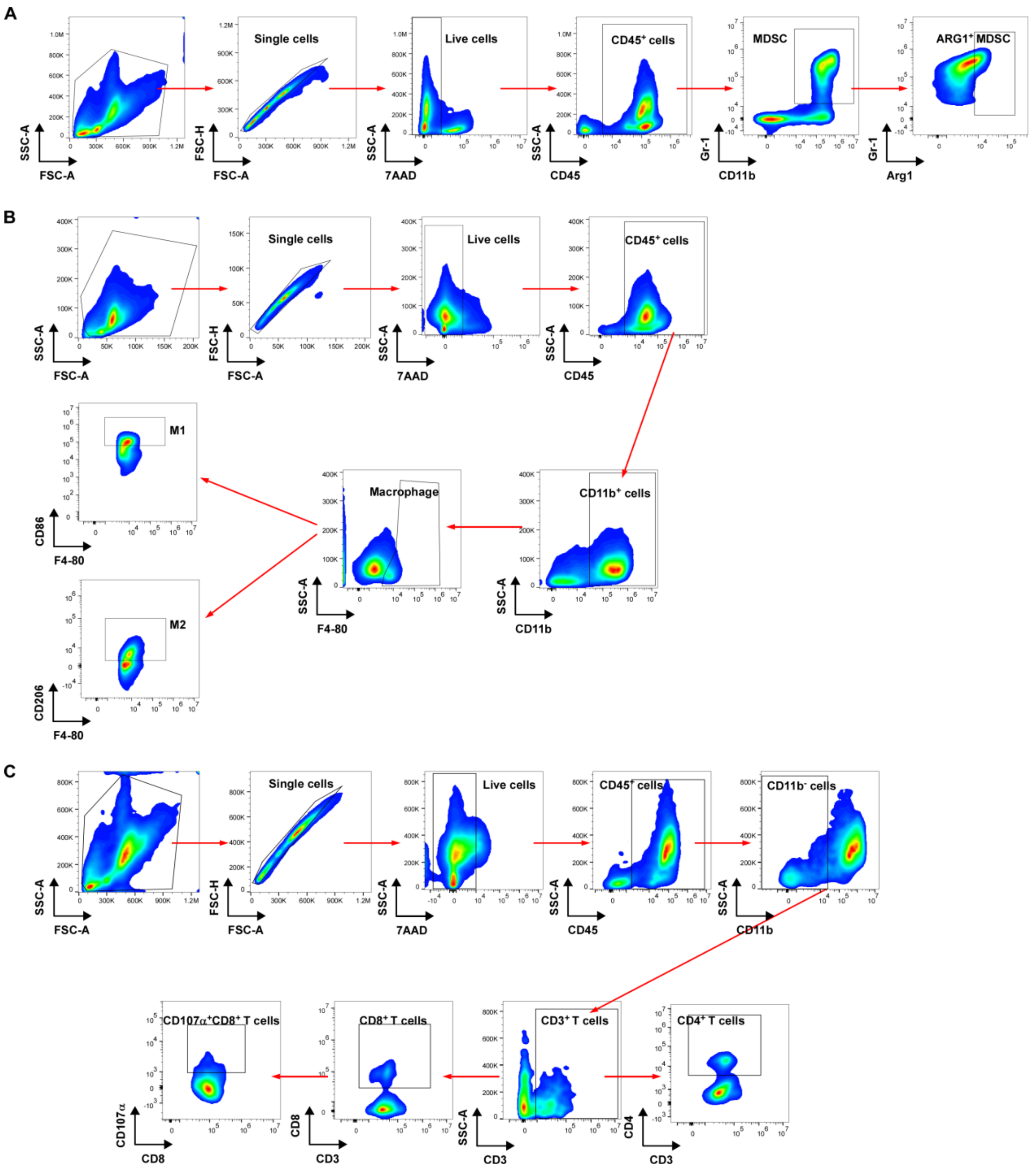

Figure S11. Flow cytometry gating strategies for the analysis of MDSCs and ARG1$^+$ MDSCs (A), M1 and M2 macrophages (B), CD4$^+$ T cells, CD8$^+$ T cells, and CD107α$^+$CD8$^+$ T cells (C) in the tumor microenvironment.

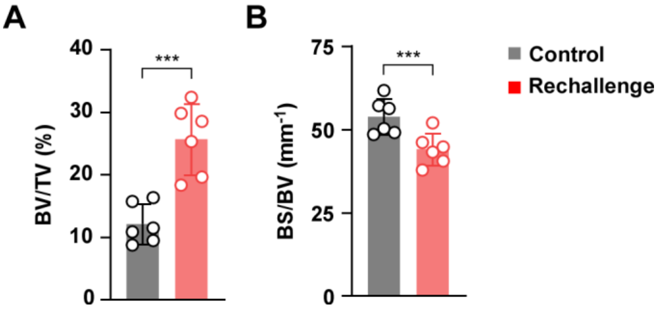

Figure S12. (A, B) Quantitative analysis of bone parameters in intratibial lesions (n = 6 per group). BV/TV, bone/tissue volume ratio. BS/BV, bone surface/volume ratio. ***, $p < 0.001$.

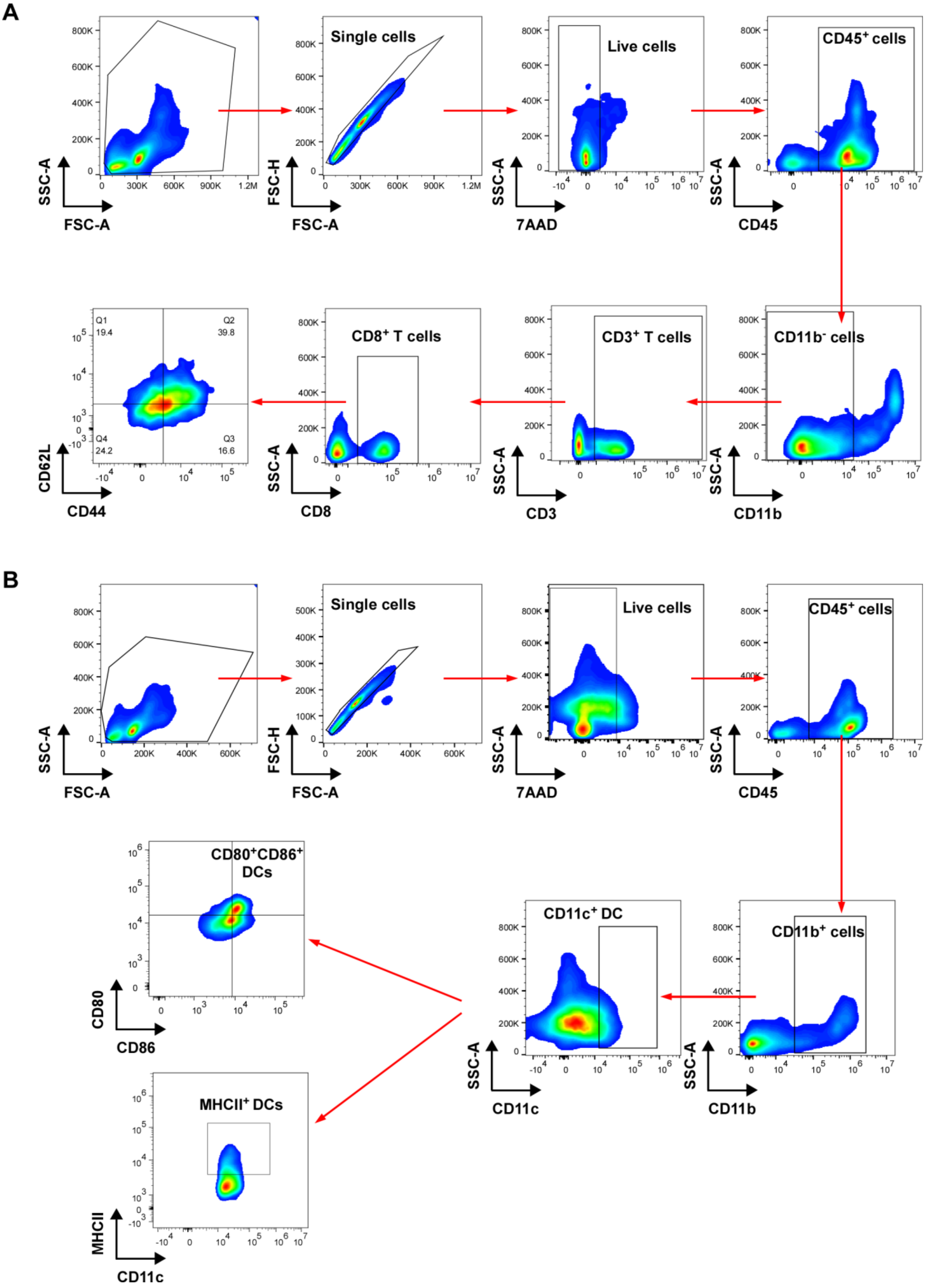

Figure S13. Flow cytometry gating strategies for the analysis of splenic T cells (A) and DC cells (B) in Figure 7.

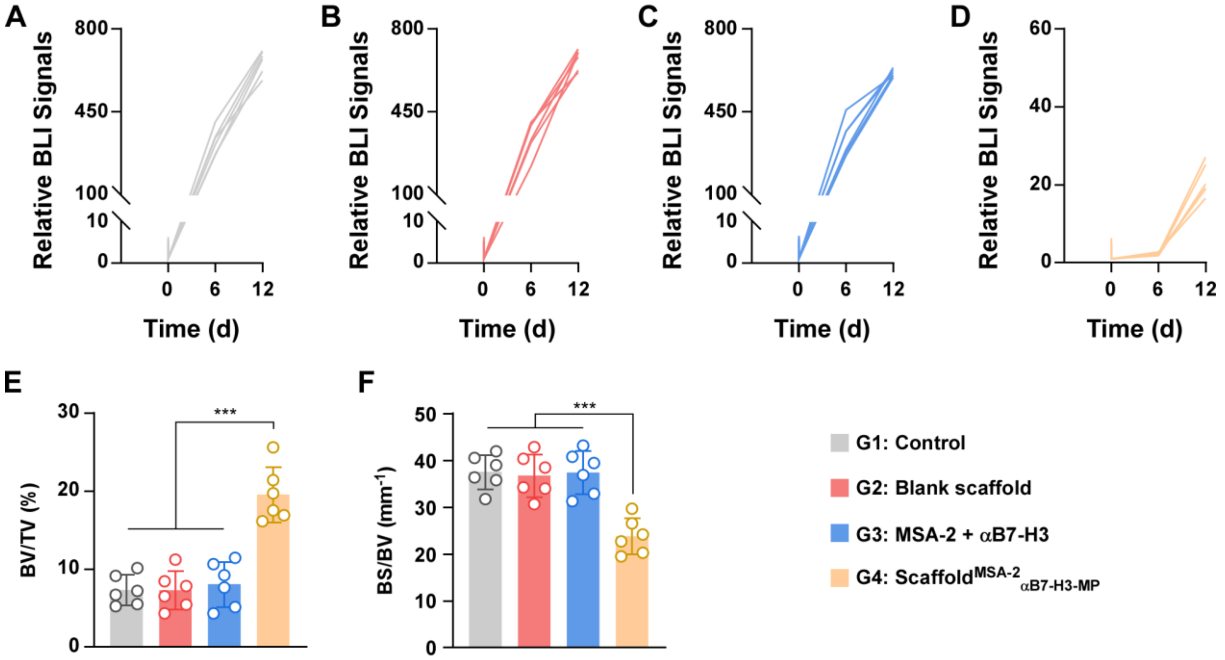

Figure S14. Evaluation of anti-tumor effects of drug-loaded GelMA scaffold in a 4T1 bone metastasis model. (A–D) Individual relative BLI signals of tibial lesions in each mouse across all experimental groups (*n* = 6 per group). (E, F) Quantitative bone analyses of BV/TV and BS/BV in intratibial lesions (*n* = 6 per group). BV/TV, bone/tissue volume ratio. BS/BV, bone surface/volume ratio. ***, $p < 0.001$.

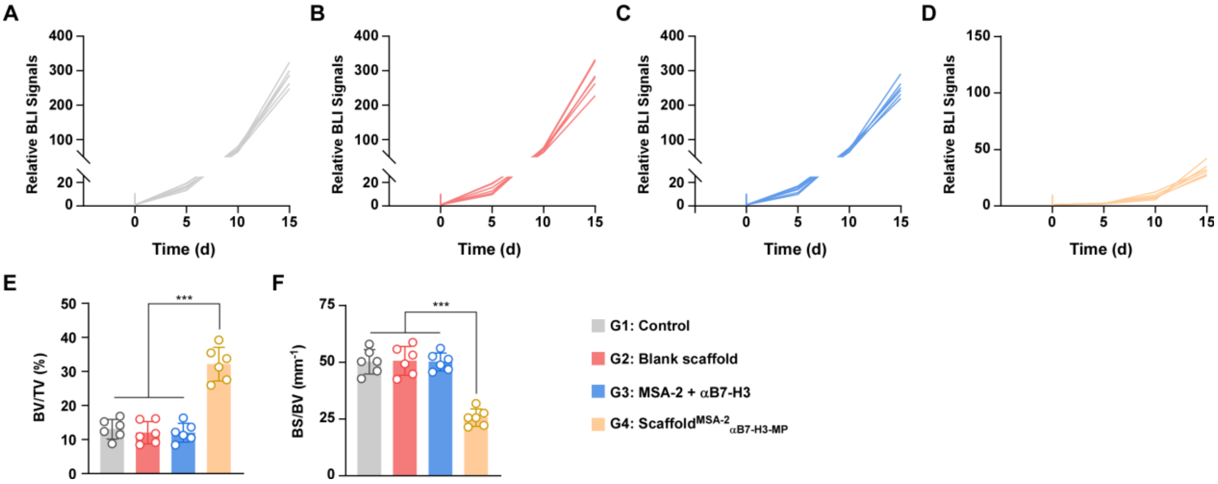

Figure S15. Evaluation of anti-tumor effects of drug-loaded GelMA scaffold in a Lewis lung carcinoma (LLC) bone metastasis model. (A–D) Individual relative BLI signals of tibial lesions in each mouse across all experimental groups (*n* = 6 per group). (E, F) Quantification of bone parameters (BV/TV and BS/BV) in intratibial lesions (*n* = 6 per group). BV/TV, bone/tissue volume ratio. BS/BV, bone surface/volume ratio. ***, $p < 0.001$.

Figure 2C

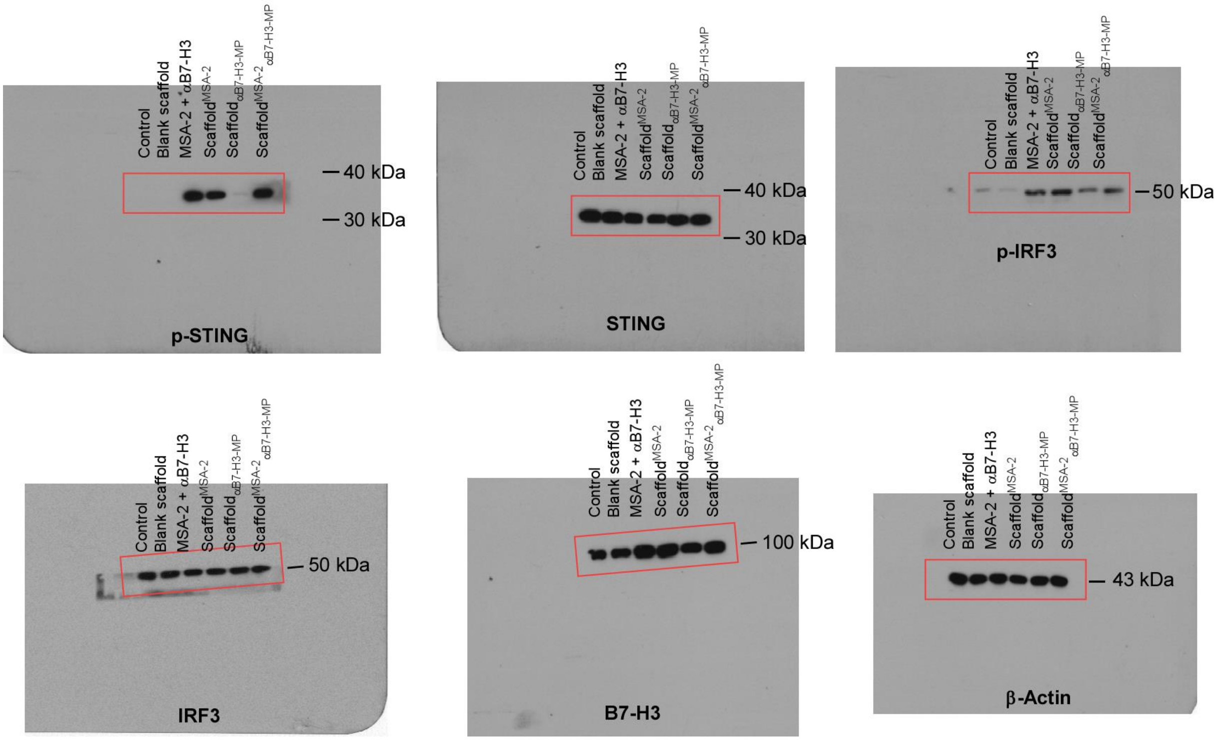

Figure S16. Uncropped western blot images for Figure 2C. Red wireframes delineate the regions cropped and displayed in Figure 2C.

Figure S4A

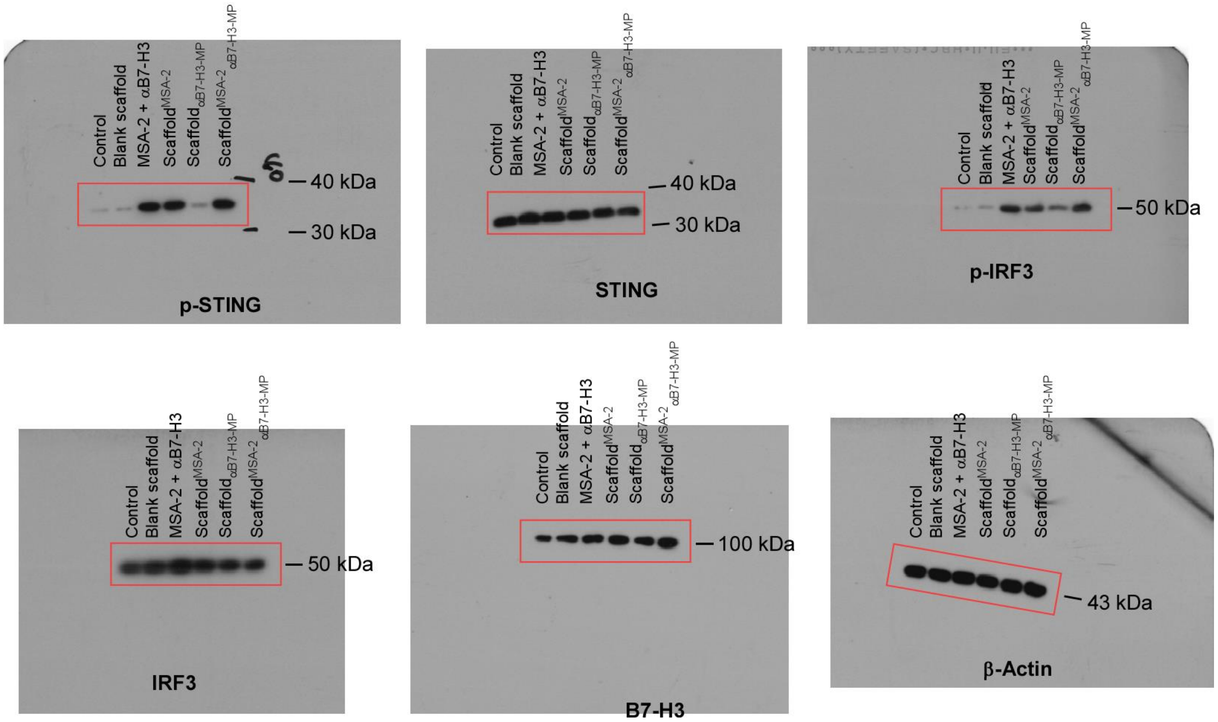

Figure S17. Uncropped western blot images for Figure S4A. Red wireframes delineate the regions cropped and displayed in Figure S4A.

Figure S4B

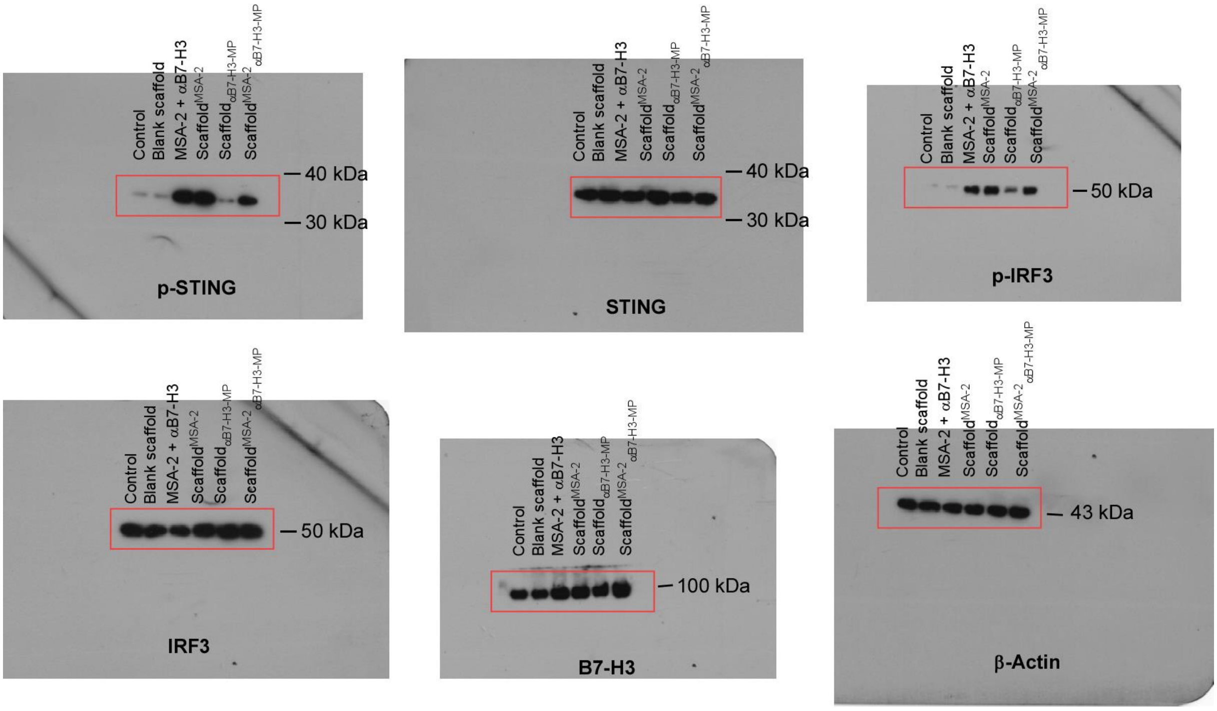

Figure S18. Uncropped western blot images for Figure S4B. Red wireframes delineate the regions cropped and displayed in Figure S4B.

Table S1. List of primers used for quantitative PCR (qPCR) analysis.

Genes	Species	Direction	Sequence (5'-3')
Arg1	Mouse	F	CATTGGCTTGCGAGACGTAGAC
		R	GCTGAAGGTCTCTTCCATCACC
Nos2	Mouse	F	GAGACAGGGAAGTCTGAAGCAC
		R	CCAGCAGTAGTTGCTCCTCTTC
Ifnb1	Mouse	F	CAGCTCCAAGAAAGGACGAAC
		R	GGCAGTGTAACTCTTCTGCAT
Actb	Mouse	F	CATTGCTGACAGGATGCAGAAGG
		R	TGCTGGAAGGTGGACAGTGAGG

Table S2. Antibodies utilized for CyTOF (Cytometry by Time-of-Flight) analysis

List	Label	Marker	Clone
1	89Y	CD45	30-F11
2	139La	CD44	IM7
3	141Pr	IL-1b	B122
4	142Nd	MHC II(I-A/I-E)	M5/114.15.2
5	143Nd	CD45R(B220)	RA3-6B2
6	144Nd	CD182(CXCR2)	SA044G4
7	145Nd	Gr-1(Ly-6G/Ly-6C)	RB6-8C5
8	146Nd	CD206(MMR)	C068C2
9	147Sm	CD80	16-10A1
10	148Nd	Ly-6C	HK1.4
11	149Sm	CD64(FcγRI)	X54-5/7.1
12	150Nd	CD14	Sa14-2
13	151Eu	CD278(ICOS)	C398.4A
14	152Sm	CD19	6D5
15	153Eu	CD274(PD-L1)	10F.9G2
16	154Sm	CD194(CCR4)	2G12
17	155Gd	CD11c	N418
18	156Gd	CD62L	MEL-14
19	157Gd	CD25(IL-2Rα)	3C7
20	158Gd	TCR γ/δ	GL3
21	159Tb	F4/80	Cl:A3-1
22	160Gd	TCR β chain	H57-597
23	161Dy	iNOS	CXNFT

24	162Dy	CD103(Integrin αE)	2E7
25	163Dy	CD172a(SIRPα)	P84
26	164Dy	CD279(PD-1)	29F.1A12
27	165Ho	IFN-γ	XMG1.2
28	166Er	Ly-6G	1A8
29	167Er	Arginase 1	E-2
30	168Er	CD49b(pan-NK cells)	DX5
31	169Tm	CD127(IL-7Rα)	A7R34
32	170Er	CD161(NK-1.1)	PK136
33	171Yb	CD69	H1.2F3
34	172Yb	CD86	GL-1
35	173Yb	Granzyme B	QA16A02
36	174Yb	CD196(CCR6)	29-2L17
37	175Lu	CD183(CXCR3)	CXCR3-173
38	176Yb	TNF-α	MP6-XT22
39	195Pt	CD3ε	145-2C11
40	197Au	CD4	RM4-5
41	198Pt	CD8a	53-6.7
42	209Bi	CD11b	M1/70
